# Engineering of ATP synthase for enhancement of proton-to-ATP ratio

**DOI:** 10.1038/s41467-025-61227-w

**Published:** 2025-07-03

**Authors:** Hiroshi Ueno, Kiyoto Yasuda, Norie Hamaguchi-Suzuki, Riku Marui, Naruhiko Adachi, Toshiya Senda, Takeshi Murata, Hiroyuki Noji

**Affiliations:** 1 Department of Applied Chemistry, Graduate School of Engineering, The University of Tokyo, Tokyo, Japan; 2 Department of Chemistry, Graduate School of Science, Chiba University, Chiba, Japan; 3 Department of Pharmacology, Graduate School of Medicine, Chiba University, Chiba, Japan; 4 Structural Biology Research Center, Institute of Materials Structure Science, High Energy Accelerator Research Organization (KEK), Ibaraki, Japan; 5 Life Science Center for Survival Dynamics, Tsukuba Advanced Research Alliance (TARA), University of Tsukuba, Ibaraki, Japan

**Keywords:** Bioenergetics, Cryoelectron microscopy, Protein design

## Abstract

F_o_F_1_-ATP synthase (F_o_F_1_) interconverts the energy of the proton motive force (*pmf*) and that of ATP through the mechanical rotation. The H^+^/ATP ratio, one of the most crucial parameters in bioenergetics, varies among species due to differences in the number of H^+^-binding c-subunits, resulting in H^+^/ATP ratios ranging from 2.7 to 5. In this study, we seek to significantly enhance the H^+^/ATP ratio by employing an alternative approach that differs from that of nature. We engineer F_o_F_1_ to form multiple peripheral stalks, each bound to a proton-conducting a-subunit. The engineered F_o_F_1_ exhibits an H^+^/ATP ratio of 5.8, surpassing the highest ratios found in naturally occurring F_o_F_1_s, enabling ATP synthesis under low *pmf* conditions where wild-type enzymes cannot synthesize ATP. Structural analysis reveals that the engineered F_o_F_1_ forms up to three peripheral stalks and a-subunits. This study not only provides valuable insights into the H^+^-transport mechanism of F_o_F_1_ but also opens up possibilities for engineering the foundation of cellular bioenergetics.

## Abstract (web summary)

F_o_F_1_-ATP synthase converts proton motive force into ATP, with the H^+^/ATP ratio typically ranging from 2.7 to 5. Here, the authors engineered this enzyme to form multiple peripheral stalks, achieving an H^+^/ATP ratio of 5.8—the highest yet reported.

## Introduction

**Fig. 1:**
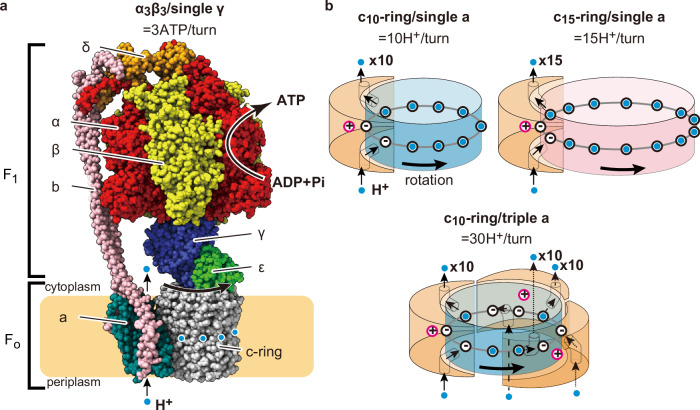
Structure of F_o_F_1_-ATP synthase and rotation mechanism of F_o_ with a different number of the c-/a-subunits. **a** Bacterial ATP synthase (from thermophilic *Bacillus* PS3) consists of F_1_ (α_3_β_3_γδε) and F_o_ (ab_2_c_10_) motors. As F_1_ has three catalytic sites, three ATP molecules are synthesized per turn of the rotor subunits (γεc_10_) against the stator subunits (α_3_β_3_δab_2_) during ATP synthesis. **b** Models of proton translocation through F_o_ coupled with the rotation of the c-ring. The highly conserved arginine residues of the a-subunit and glutamate (or aspartate) residues of c-subunits are depicted with pink and black open circles, respectively. Protons are depicted as light blue circles. In the models of c_10_-ring/single a-subunit and c_15_-ring/single a-subunit, 10 and 15 protons, equal to the number of the c-subunits, are transferred in one turn, respectively. Conversely, in the c_10_-ring and triple a-subunits model, a total of 30 protons, equal to the number of the c-subunits multiplied by the number of the a-subunits, are transferred in one turn.

F_o_F_1_-ATP synthase (F_o_F_1_) is a ubiquitous enzyme found in the membranes of mitochondria, chloroplasts and bacteria. It synthesizes ATP from ADP and inorganic phosphate (Pi) coupled with proton translocation across membranes along the proton motive force (*pmf*)^1–3^. F_o_F_1_ is a unique molecular motor complex composed of two rotary molecular motors: F_1_ and F_o_^1,2^. Bacterial F_o_F_1_ exhibits the simplest subunit composition of a_1_b_2_c_*x*_ (*x* varies among species) for F_o_ and α_3_β_3_γ_1_δ_1_ε_1_ for F_1_^4–6^ (Fig. 1a). F_o_ is a membrane-embedded molecular motor driven by *pmf*. When protons are translocated through the proton pathway in F_o_ along *pmf*, the multimeric rotor ring composed of c-subunits (termed c-ring) rotates against stator ab_2_ (Fig. 1a, b). F_1_ is the catalytic core domain of F_o_F_1_ for ATP synthesis and hydrolysis^7^. When isolated from F_o_, F_1_ acts as an ATP-driven molecular motor that rotates the γε rotor complex against the catalytic α_3_β_3_ stator ring coupled with ATP hydrolysis^8^. These two motors are coupled via two stalks: the peripheral stalk, composed of the b_2_ dimer stalk and the δ subunit. The central rotor stalk is composed of a γε complex and a c-ring. Under ATP synthesis conditions where *pmf* is sufficient and the rotational torque of F_o_ exceeds that of F_1_, F_o_ rotates the γε complex in F_1_ in the reverse direction of ATP hydrolysis, driving the ATP synthesis reaction on the α_3_β_3_ ring^9,10^. Conversely, when the torque of F_1_ exceeds that of F_o_, F_1_ rotates the c-ring in F_o_, forcing F_o_ to pump protons in the reverse direction, thereby generating *pmf*. Thus, F_o_F_1_ interconverts the *pmf* and chemical potential of ATP hydrolysis through mechanical rotation.

Considering the Gibbs free energy of this coupling reaction ($\Delta G^{\prime}$),

1 $${{{\rm{A}}}}{{{\rm{D}}}}{{{\rm{P}}}}+{{{\rm{P}}}}{{{\rm{i}}}}+n{{{{\rm{H}}}}}_{{{{\rm{i}}}}{{{\rm{n}}}}}^{+}\rightleftharpoons {{{\rm{A}}}}{{{\rm{T}}}}{{{\rm{P}}}}+{{{{\rm{H}}}}}_{2}{{{\rm{O}}}}+n{{{{\rm{H}}}}}_{{{{\rm{o}}}}{{{\rm{u}}}}{{{\rm{t}}}}}^{+}$$

$$\Delta G^{\prime}$$ is givens:

2 $$\Delta G^{\prime}=\Delta {G}_{{{{\rm{ATP}}}}}^{{\prime} }-nF\cdot pmf$$

where $\Delta {G}_{{{{\rm{ATP}}}}}^{{\prime} }$ is the Gibbs free energy of ATP synthesis, $F$ is Faraday’s constant, $n$ is the H^+^/ATP ratio, which is defined as the number of protons translocated through F_o_ coupled with a single turnover of ATP synthesis on F_1_. Hence, the following conditions must be satisfied to drive ATP synthesis:

3 $$\Delta {G}_{{{{\rm{ATP}}}}}^{{\prime} } < nF\cdot pmf$$

Thus, the H^+^/ATP ratio is the critical factor in determining the lower limit of *pmf* required for ATP synthesis, given that $\Delta {G}_{{{{\rm{ATP}}}}}^{{\prime} }$ does not largely vary among species. The H^+^/ATP ratio is principally defined by the ratio of the reaction stoichiometry of F_o_ per turn of the rotor complex to that of F_1_, and the ratio of H^+^/turn of F_o_ to ATP/turn of F_1_. All the F_1_s studied so far, without exception, have three catalytic β subunits and couple three reactions of ATP hydrolysis/synthesis per turn, defining the ATP/turn ratio as 3^10^. The reaction stoichiometry of F_o,_ H^+^/turn varies among species due to differences in the number of c-subunits in the c-ring. According to the half-channel model, supported by recent structural studies, the H^+^ pathway in F_o_ is formed by the c-ring and the a-subunit, which has two half-channels exposed to the periplasmic or cytoplasmic side of the membrane^11–13^. During ATP synthesis, H^+^ from the periplasmic solution enters the half-channel exposed to the periplasmic space and is transferred to one of the c-subunits in the c-ring. Following one revolution of the c-ring, H^+^ is released into the cytoplasmic solution through the opposite half-channel (Fig. 1b, Supplementary Fig. 1). Thus, the half-channel model assumes that the stoichiometry of H^+^/turn is determined by the number of c-subunits.

The number of c-subunits in the c-ring in F-type ATP synthases ranges from 8 to 15, depending on the species^14–16^. When assuming perfect energy coupling between F_1_ and F_o_, the H^+^/ATP ratio should vary between 2.7 and 5.0 among the species. Various groups have attempted to experimentally determine the H^+^/ATP ratio from the biochemical measurements of the thermodynamic equilibrium point where the *pmf* and ${\Delta G}_{{{{\rm{ATP}}}}}^{{\prime} }$ are balanced. The *Bacillus* PS3 F_o_F_1_, with a c_10_-ring, has been reported to show good agreement with the structurally expected H^+^/ATP ratio of 3.3^17^. F_o_F_1_s from *E. coli* and yeast mitochondria, both of which also have the c_10_-ring, exhibit slightly different H^+^/ATP ratios: 4.0 ± 0.3^18^ and 2.9 ± 0.2^19^, respectively. For spinach chloroplast F_o_F_1_ with the c_14_-ring, two independent studies reported smaller values for the H^+^/ATP ratio: 4.0 ± 0.2^18^ and 3.9 ± 0.3^19^, which are lower than the expected value of 4.7. Thus, the experimentally determined H^+^/ATP ratios are close to, but not always identical to, the structurally expected values, varying within a narrow range of 3 to 4.

The H^+^/ATP ratio of F_o_F_1_ is one of the most critical parameters in the bioenergetic system of cells, which defines the energy cost of ATP synthesis and the threshold *pmf* required for ATP synthesis (see Eq. 3)^14,17^. Since $\Delta {G}_{{{{\rm{ATP}}}}}^{\prime}$ does not largely differ across organisms, F_o_F_1_ with a higher H^+^/ATP ratio can synthesize ATP even at a lower *pmf*. In fact, alkaliphilic bacteria living in highly alkaline environments and photosynthetic organisms that grow under light-limiting conditions have a c-ring with a large number of c-subunits^20–22^. This is thought to be an evolutionary adaptation that allows stable ATP synthesis under low and/or unstable *pmf* conditions^22,23^. Thus, organisms may have optimized the H^+^/ATP ratio through evolution by tuning the stoichiometry of the c-ring to meet their energetic requirements.

Conversely, when reconsidering the half-channel mechanism, we can assume that the stoichiometry of H^+^/turn in F_o_ is determined not only by the number of c-subunits but also by the number of a-subunits (Fig. 1b, bottom). In particular, the F_o_ structures solved so far show that a large portion of the c-ring is exposed to the lipid bilayer, suggesting the possibility of accommodating one or two additional a*-*subunits, although F_o_F_1_ with multiple a-subunits has not yet been identified.

Here, we explore the possibility of doubling or tripling the H^+^/ATP ratio of F_o_F_1_ by increasing the number of a-subunits in F_o_, rather than increasing the stoichiometry of the c-ring as observed in nature.

## Results

### Design for multiple peripheral stalks

**Fig. 2:**
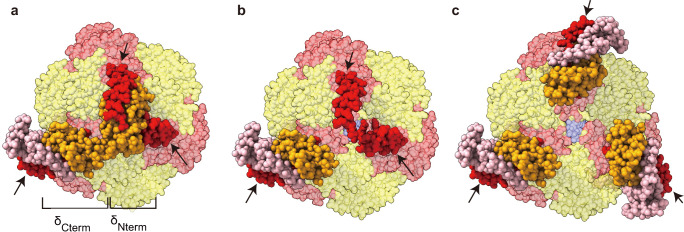
Design strategy to multiply peripheral stalks. Top views of *Bacillus* PS3 F_o_F_1_ from the cytoplasm (PDB ID: 6N2Y). The α (red), β (yellow), γ (blue), δ subunits (orange), and the b_2_ stalk (pink) are shown. The non-transparent red parts indicated by the arrows represent the N-terminal region (residues 2–30) of the α subunit. **a** Asymmetric interactions between the δ and α subunits. The single δ subunit interacts with the three α subunits, occupying the central concavity of the α_3_β_3_ subcomplex. **b** Model structure after deletion of the N-terminal domain (residues 2–104) of the δ subunit. The central concavity is exposed, and two of three α subunits are unoccupied. **c** Model structure after each of the N-terminus of the three α subunits is fused to the C-terminal domain (residues 105–178) of the δ subunit.

So far, all ATP synthases have a single copy of the a-subunit per F_o_F_1_ complex. Considering that the a-subunit is tightly bound to the membrane portion of the peripheral stalk, the key factor for multiplying the a-subunits should be the structural mechanism that limits the number of peripheral stalks. The peripheral stalk of the b_2_ dimer extends from the membrane to the upper surface of the α_3_β_3_ subcomplex of F_1_, binding to the δ subunit. The δ subunit binds to the top of the α_3_β_3_ subcomplex, associating with the N-terminal regions of the three α subunits. One α subunit interacts with the C-terminal region of the δ subunit, while the other two interact with the N-terminal region of the δ subunit, as indicated by the arrows in Fig. 2a. Since the N-terminal domain of the δ subunit is located in the central concavity of the α_3_β_3_ ring, occupying the pseudo-threefold symmetry axis of the α_3_β_3_ ring, it is reasonable to assume that the N-terminal domain of the δ subunit disrupts the pseudo-threefold symmetry, limiting the stoichiometry of the peripheral stalk to one (Fig. 2a). We hypothesized that by removing the N-terminal domain of the δ subunit (Fig. 2b), it becomes possible to accommodate a truncated δ subunit on each α subunit (Fig. 2c). A possible concern regarding the truncation is that the truncated δ subunit (δ_ΔN_) does not form a stable complex with the α_3_β_3_ ring. Therefore, we designed the δ_ΔN_-α fusion construct of *Bacillus* PS3 F_o_F_1_, where the C-terminus of the δ subunit was genetically fused to the N-terminus of the α subunit. In addition, the inhibitory C-terminal domain of the ε subunit was removed to enhance enzymatic activity^17,24^. In this study, *Bacillus* PS3 F_o_F_1_-ε_ΔC_ was used as a wild-type F_o_F_1_ for comparison.

### SDS-PAGE analysis of subunit stoichiometry

**Fig. 3:**
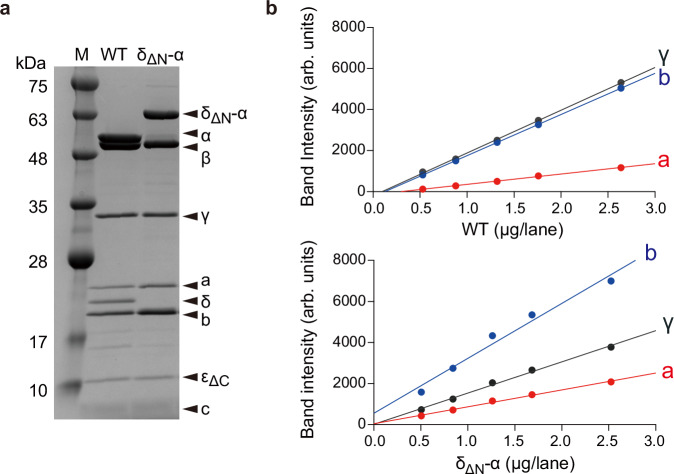
Subunit stoichiometry of δ_ΔN_-α fused F_o_F_1_. **a** SDS-PAGE analysis of the purified wild-type (WT) *Bacillus* PS3 F_o_F_1_ and the δ_ΔN_-α fused F_o_F_1_. Each sample was derived from a single purification batch. 3 μg of F_o_F_1_ was loaded in each lane. The molecular masses of the δ_ΔN_-α, α, β, γ, a, δ, b, ε_ΔC_, and c-subunits are 63, 55, 53, 32, 26, 20, 19, 9, and 7 kDa, respectively. The experiment was independently repeated three times with similar results. **b** The band intensity vs total protein amount. A single series of diluted F_o_F_1_ from the same purification batch was loaded into a gel and subjected to SDS-PAGE analysis. The band intensity of each subunit was plotted against the total amount of F_o_F_1_ loaded for SDS-PAGE analysis. The plots were fitted with a linear function. The slopes for γ (black), a- (red), and b-subunits (blue) were determined to be 2082, 502, and 2004 (arb. units/µg) for the WT F_o_F_1_, and 1521, 821, and 2675 (arb. units/µg) for the δ_ΔN_-α fused F_o_F_1_, respectively. By normalizing the slopes of the a- and b-subunits to the slope of the γ subunit of each F_o_F_1_, the stoichiometries of the a- and b-subunits of the δ_ΔN_-α fused F_o_F_1_ were estimated to be 2.2 ( = (821/1521)/(502/2082)) and 1.8 ( = (2675/1521)/(2004/2082)) times higher than those of the wild-type F_o_F_1_, respectively.

The δ_ΔN_-α fused F_o_F_1_ was purified following a previously reported procedure for the wild-type *Bacillus* PS3 F_o_F_1_^17^. To estimate the subunit stoichiometry, the δ_ΔN_-α fused F_o_F_1_ was analyzed by SDS-PAGE with the wild-type F_o_F_1_ for comparison (Fig. 3a, b). The δ_ΔN_-α fused F_o_F_1_ lacked δ and α, and the δ_ΔN_-α fusion appeared above the band position for the α subunit (Fig. 3a). The δ_ΔN_-α fused F_o_F_1_ retained the complete set of subunits. Then, we estimated the subunit stoichiometry of the a- and b-subunits in the δ_ΔN_-α fused F_o_F_1_ by using the γ subunit as the internal reference in comparison with the wild-type (Fig. 3b, Supplementary Fig. 2). In the wild-type, the b-subunit exhibited a band intensity comparable to that of the γ subunit, whereas in the δ_ΔN_-α fused F_o_F_1_, the b-subunit showed higher signals relative to the γ subunit. Similarly, the δ_ΔN_-α fused F_o_F_1_ exhibited a higher band intensity for the a-subunit compared to the wild type, indicating that the δ_ΔN_-α fused F_o_F_1_ increases the stoichiometry of the a- and b-subunits. For a more quantitative estimation, we plotted calibration lines for the wild-type and mutant subunits and standardized the lines using the γ subunit calibration lines as the internal control (Fig. 3b). We then determined the stoichiometries of the a- and b-subunits in the δ_ΔN_-α fused F_o_F_1_ by comparing them to the wild-type. The estimated stoichiometries of the a- and b-subunits were 2.2 and 1.8 times higher than those of the wild-type F_o_F_1_, respectively (see Fig. 3b legend). Thus, it was confirmed that the δ_ΔN_-α fused F_o_F_1_ has multiple, two on average, peripheral stalks and a-subunits.

### Functional analysis of the H^+^/ATP ratio

**Fig. 4:**
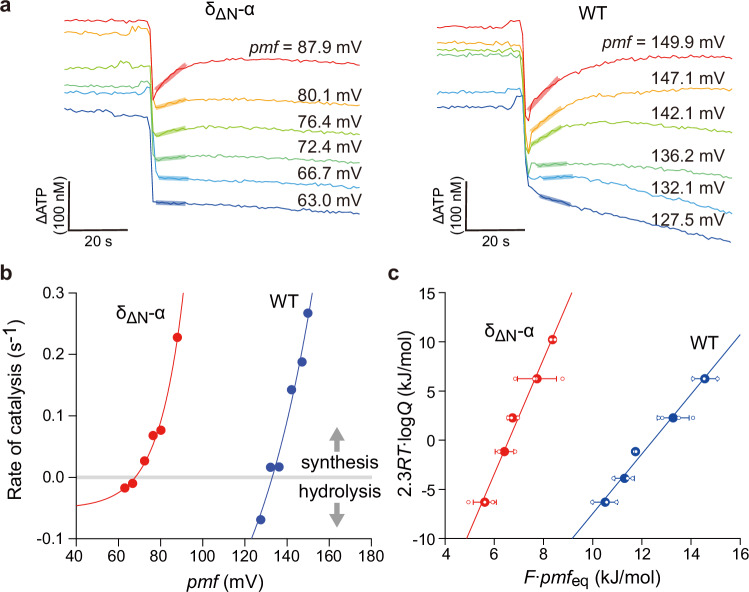
Functional analysis for the determination of the H^+^/ATP ratio. **a** Time courses of the ATP synthesis/hydrolysis activity of the reconstituted proteoliposomes (PLs) at different *pmf*. ATP synthesis reaction was measured using the luciferin/luciferase system. The reaction quotient, *Q* was 2.5; [ATP] = 500 nM, [ADP] = 20 µM, [Pi] = 10 mM. The rate of catalysis was determined from the initial slopes (bold lines). **b** The ATP synthesis/hydrolysis rates determined from (**a**) were plotted against *pmf*. The data points were fitted with an exponential function for the determination of the equilibrium *pmf, pmf*_eq_, as the interception of the *x* axis. **c** Determination of the H^+^/ATP ratio. The mean (filled circles) and the SD of $F\cdot {{pmf}}_{{eq}}$ values were determined from 3 to 4 independent biological replicates (open circles) at each *Q* condition using different batches of purified enzymes, and $2.3{RT}\cdot \log Q$ values were plotted against the corresponding $F\cdot {{pmf}}_{{eq}}$ values according to Eq. (5). Sample sizes (*n*) from left to right are: 3, 3, 3, 4, 3 (WT) and 4, 3, 3, 4, 3 (δ_ΔN_-α). Each line represents a linear regression fit to the dataset obtained from each F_o_F_1_.

We attempted to determine the H^+^/ATP ratio of the δ_ΔN_-α fused F_o_F_1_ through biochemical measurements of the thermodynamic equilibrium between *pmf* and $\Delta {G}_{{{{\rm{ATP}}}}}^{,}$, as previously reported^17^. Firstly, we prepared the F_o_F_1_-reconstituted proteoliposomes (PLs) and incubated them in an acidic buffer. The PLs were injected into the base assay medium to initiate ATP synthesis. The ATP synthesis/hydrolysis activity was monitored with the luciferin/luciferase assay system under various *pmf* conditions, with a given reaction quotient, *Q* $(=\left[{\mbox{ATP}}\right]/\left(\left[{\mbox{ADP}}\right]\cdot \left[{\mbox{Pi}}\right]\right))$. Figure 4a shows the time courses of the assay, in which the initial rate was determined. Figure 4b shows the reaction rates plotted against the *pmf* when *Q* = 2.5. The δ_ΔN_-α fused F_o_F_1_ was shown to catalyze the ATP synthesis reaction even at low *pmf*, at which wild-type F_o_F_1_ is unable to synthesize ATP. For a more quantitative analysis, the data points were fitted with an exponential function to determine the equilibrium *pmf* (*pmf*_eq_), where the torques of F_1_ and F_o_ are balanced and the net reaction rate is zero. At the condition of Fig. 4b where *Q* = 2.5, *pmf*_eq_ was determined to be 68 mV for the δ_ΔN_-α fused F_o_F_1_ and 133 mV for the wild-type. Thus, the minimum *pmf* for ATP synthesis was halved for the δ_ΔN_-α fused F_o_F_1_, suggesting that the functional H^+^/ATP ratio of the δ_ΔN_-α fused F_o_F_1_ is also doubled, in agreement with SDS-PAGE analysis. For further confirmation, we determined the *pmf*_eq_ at various *Q* values (Supplementary Fig. 3). Under all conditions, the *pmf*_eq_ of the δ_ΔN_-α fused F_o_F_1_ was nearly half that of the wild-type.

For the comprehensive analysis of the functional H^+^/ATP ratio, Eq. (2) was transformed as below,

4 $$\Delta {G}^{{\prime} }=\Delta {G}_{{ATP}}^{{\prime} }-{nF}\cdot {pmf}=\Delta {G}_{{ATP}}^{0{\prime} }+2.3{RT}\cdot \log Q-{nF}\cdot {{pmf}}_{{eq}}=0$$

5 $$2.3{RT}\cdot \log Q=-\Delta {G}_{{ATP}}^{0{\prime} }+{nF}\cdot {{pmf}}_{{eq}}$$

where $\Delta {G}_{{ATP}}^{0,}$ is the Gibbs free energy of ATP synthesis under the biochemical standard state, *R* and *T* are the gas constant and absolute temperature, respectively. Here, *pmf*_eq_ values were experimentally determined under defined *Q* conditions. The other values are constant. Therefore, when $2.3{RT}\cdot \log Q$ is plotted against $F\cdot {{pmf}}_{{eq}}$, *n* (= the H^+^/ATP ratio) is determined as the slope of the data points. In addition, ${G}_{{ATP}}^{0,}$ is determined as the interception of the y axis. As shown in Fig. 4c, the δ_ΔN_-α fused F_o_F_1_ exhibited a significantly steeper slope compared to the wild-type F_o_F_1_. From the linear fitting, the H^+^/ATP ratio was determined to be 5.8 ± 0.4 and 3.0 ± 0.2 (fitted value ± SE of the fit) for the δ_ΔN_-α fused F_o_F_1_ and wild-type, respectively. Although the H^+^/ATP ratio of the wild-type is slightly lower than the structurally expected value of 3.3 and the reported value (3.3 ± 0.1)^17^, δ_ΔN_-α fused F_o_F_1_ was shown to double the H^+^/ATP ratio, in close agreement with the subunit stoichiometry analysis from SDS-PAGE. This agreement suggests that the δ_ΔN_-α fused F_o_F_1_ has two functional a-subunits in the ensemble average. From the interception of the y axis, the $\Delta {G}_{{{{\rm{ATP}}}}}^{0,}$ values are determined to be 38 ± 3 kJ mol^−1^ (fitted value ± SE of the fit) for both. This value shows a fine agreement with the reported values for *Bacillus* PS3 F_o_F_1_ (39 ± 1 kJ mol^−1^)^17^, *E. coli* (38 ± 3 kJ mol^−1^)^18^, yeast (36 ± 3 kJ mol^−1^)^19^, and chloroplasts (38 ± 3 and 37 ± 3 kJ mol^−1^)^18,19^, supporting the validity of the experiment.

### Cryo-EM structural analysis

**Fig. 5:**
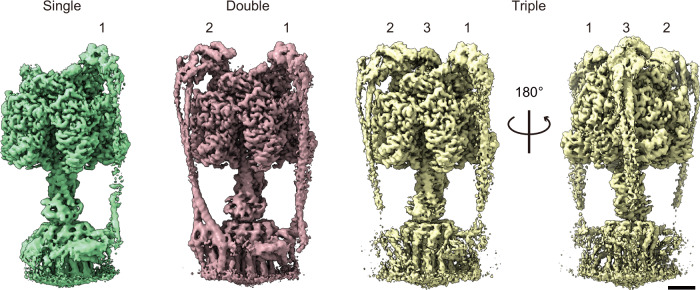
Cryo-EM maps of F_o_F_1_ with multiple peripheral stalks. The composite cryo-EM maps of F_o_F_1_ with single, double, and triple peripheral stalks. The number represents the position of each peripheral stalk. Scale bar, 25 Å.

We determined the structure of the δ_ΔN_-α fused F_o_F_1_ by single-particle cryo-EM analysis. The purified δ_ΔN_-α fused F_o_F_1_ in detergent was applied to EM grids, frozen in liquid ethane, and imaged with 300 kV cryo-EM followed by single-particle analysis using cryoSPARC. The cryo-EM map was obtained by ab initio 3D reconstruction and classification, followed by refinement with C1 symmetry. Since the rotor complex in F_o_F is oriented at one of the three catalytic dwell angles relative to the stator ring and peripheral stalk, alignment against the central core complex, including the rotor complex, revealed three distinct positions of the peripheral stalk, each separated by 120°, as observed in previous reports^25,26^. Map structure classification was performed by masking the peripheral-stalk positions, confirming the presence or absence of the peripheral stalk at the masked position (Supplementary Fig. 4). This classification was conducted for each stalk position: Stalks 1, 2, and 3. Thus, the map structure was classified into eight sub-classes. The overall resolution was 2.5–3.2 Å (Supplementary Fig. 4). While the wild-type F_o_F_1_ contains only a single peripheral stalk, this structural classification confirmed that some fractions of the particles contained multiple peripheral stalks (Fig. 5 and Supplementary Fig. 4). A significant fraction of the particles, however, had either no peripheral stalk or only a single one. The percentages of F_o_F_1_ structures with 0, 1, 2, and 3 peripheral stalks, as determined from the 3D classification, were 15, 51, 26, and 8%, respectively. Because of the lower resolution of the F_o_ region, focused refinement with F_o_ was conducted by masking the F_o_ region. The refined structure achieved resolutions of 3.5–6.6 Å, confirming that the peripheral stalk always accompanies F_o_ a*-*subunits (Supplementary Fig. 4). Thus, the percentage of F_o_F_1_ with 0, 1, 2, and 3 a-subunits should correspond to that for peripheral stalks. The fractions of F_o_F_1_ with multiple a-subunits were small, and the ensemble average of peripheral stalk was only 1.26 per molecule, which is evidently lower than the expected value of ~2 per molecule based on the subunit stoichiometry analysis and the functional analysis of H^+^/ATP ratio. Although the exact reason for this discrepancy is unclear, it is highly likely that the peripheral stalk and the a-subunit dissociated due to the meniscus force and/or the interaction with the air/water interface during cryo-EM grid preparation^27^.

### Interaction between the peripheral stalk and the δ_ΔN_-α fusion

Among the eight sub-classes, three structures contained a single copy of the peripheral stalk, each located at position Stalk 1, Stalk 2, or Stalk 3. These structures fit well with the reported three rotational state structure of the wild-type *Bacillus* PS3 F_o_F_1_^25^, respectively (Supplementary Fig. 5), indicating the structural integrity of the binding of the b_2_ dimer to the F_1_ part via δ_ΔN_-α fusion. Slight differences were observed at the top of the F_1_ headpiece and on the side of the central stalk because the δ_ΔN_-α fused F_o_F_1_ lacks the N-terminal region of the δ subunit and the C-terminal helix of the ε subunit. The maps of the double- and triple-stalk F_o_F_1_ were compared with the corresponding maps of single-stalk F_o_F_1_, respectively (Supplementary Fig. 6). These maps were well fitted, indicating that multiple stalks have no significant structural constraints on the whole structure of F_o_F_1_.

**Fig. 6:**
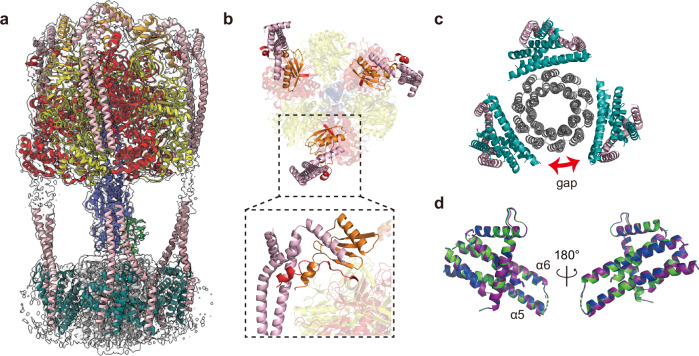
Atomic models of F_o_F_1_ with triple peripheral stalks. **a** Composite map and atomic models for the F_1_ and F_o_ regions of the triple-stalk F_o_F_1_. The middle regions of the b-subunits could not be modeled due to the lack of clear density. **b** The top view of the structure. The close-up view shows the side view of the interaction between δ_ΔN_-α fusion and the b_2_ dimer. **c** The view from the bottom of the structure shown in (**a**). **d** The superposition of the three a*-*subunits (green, purple, and blue) in the triple-stalk F_o_.

The atomic models for triple-stalk F_o_F_1_ are shown in Fig. 6. The structure of the δ_ΔN_-α fusion region was well resolved, providing atomic details of its binding site with the b_2_ dimer (Fig. 6a, b). As designed, the three binding sites were almost identical, with structures similar to those found in wild type *Bacillus* PS3 F_o_F_1_^25^ and other ATP synthases^28^ (Supplementary Fig. 7a). These observations reveal that the three b_2_ dimers are incorporated via the canonical interaction with δ_ΔN_-*a* fusion, suggesting the integrity of the peripheral stalks of the δ_ΔN_-α fused F_o_F_1_.

### Structure of the a-subunits

The structure and the position of the a-subunits of the triple-stalk F_o_F_1_ were investigated by comparing them with those in the wild-type *Bacillus* PS3 F_o_F_1_^25^ (Fig. 6c and Supplementary Fig. 7b). The spatial intervals between the a-subunits are not perfectly symmetric because of the symmetry mismatch between the ring structures of F_1_ and F_o_, that is, threefold versus tenfold. Each a-subunit interacted with three neighboring c-subunits, forming an a_1_c_3_ unit. Therefore, a total of nine c-subunits interacted with the a-subunits, leaving the remaining c-subunit at the open position (gap) between two a*-*subunits (Fig. 6c). As a result, the three a-subunits were not positioned exactly 120° apart from each other. The asymmetric positioning of the a-subunits is in good agreement with that suggested by the three states of the wild-type *Bacillus* PS3 F_o_F_1_^25^. When the a-subunits in the triple-stalk F_o_F_1_ were compared to each other, Cα-RMSDs were 0.3–0.5 Å (Fig. 6d). In addition, the Cα-RMSDs estimated by superimposition of each a-subunit in the triple-stalk F_o_F_1_ with that in the corresponding state of the wild-type F_o_F_1_ were 0.9–1.0 Å. Moreover, superimposing the a_1_*c*_3_ units in the same manner yielded Cα-RMSDs of 1.0–1.2 Å (Supplementary Fig. 7c). Thus, at the current resolution, the overall structures of the three a-subunits in the triple-stalk F_o_F_1_ were essentially identical to each other and closely resembled that of the wild-type *Bacillus* PS3 F_o_F_1_. This suggests that all a-subunits are functional, which is consistent with the aforementioned biochemical analyses showing the enhanced H^+^/ATP ratio.

### Structure of F_1_ part

The structure of the F_1_ portion of the triple-stalk F_o_F_1_ was investigated by comparing it with previously reported structures. The F_1_ structure was found to be very similar to that of the *Bacillus* PS3 F_o_F_1_-ε_ΔC_ under uni-site catalysis conditions^26^, with one β subunit bound to ADP in a closed conformation (${{{{\rm{\beta }}}}}_{{{{\rm{TP}}}}}^{C}$) and two β subunits without bound nucleotide adopting open conformations (${{{{\rm{\beta }}}}}_{{{{\rm{E}}}}}^{O}$, ${{{{\rm{\beta }}}}}_{{{{\rm{DP}}}}}^{O}$) (Supplementary Fig. 8a, b). The purified δ_ΔN_-α fused F_o_F_1_ was prepared in nucleotide-free conditions. Therefore, it is highly likely that the bound ADP was endogenous. Notably, at a low-density threshold, weak map density was observed at the outer periphery of ${{{{\rm{\beta }}}}}_{{{{\rm{TP}}}}}^{C}$, which can be well fitted with the open β conformation of the nucleotide-depleted F_1_ in the *Bacillus* PS3 F_o_F_1_-ε_ΔC_^26^ (Supplementary Fig. 8c). This suggests that the cryo-EM map includes two conformations: closed and open. Other sub-class structures also exhibited similarly mixed maps.

## Discussion

The present study provides insights into the design principles of ATP synthases using an engineering approach. Firstly, the δ subunit is the factor that defines the number of peripheral stalks. In this study, the N-terminal domain of δ, which breaks the structural pseudo-threefold symmetry by binding to the position on the symmetry axis, was deleted, and the C-terminal domain of δ was genetically fused to the N-terminal of the α subunit. As a result, up to three peripheral stalks were incorporated into the F_o_F_1_ complex. Structural analysis using cryo-EM revealed that the δ_ΔN_-α fusion and the b_2_ dimer adopted the canonical binding structure observed in the wild-type F_o_F_1_, except for the missing N-terminal domain of the δ subunit. This observation clearly shows that the N-terminal domain of the δ subunit determines the number of peripheral stalks per F_o_F_1_, restricting it to one by disrupting the structural symmetry.

Next, the number of a*-*subunits is determined based on the number of peripheral stalks. While cryo-EM analysis showed that some molecules lost peripheral stalks due to the detachment of the b_2_ dimer, the observed peripheral stalks always remained bound to the a*-*subunits, indicating stable binding between the a-subunit and the b_2_ dimer. Thus, the number of peripheral stalks determines the number of a-subunits. The structure of the triple-stalk F_o_F_1_ clearly showed that the c_10_ ring can accommodate up to three a-subunits, but not more than four due to spatial constraints. Considering that each a*-*subunit can interact with three c-subunits, accommodating four or more a-subunits in F_o_F_1_ would require the c-ring consisting of more than 12 c-subunits, along with further engineering on the F_1_ part, such as introducing a peripheral interaction site at the N-terminus of the β-subunit.

Another important finding of this study is the functional independence of the a-subunit, at least in terms of the coupling stoichiometry of H^+^ (see below). In the triple-stalk F_o_F_1_, all three a-subunits interacted with the c-ring. In addition, the structural features of the interaction agreed well with those observed in the wild-type F_o_F_1_ structures. This suggests that each of the three a-subunits is functional. SDS-PAGE analysis revealed that the samples used in this study had an average of two peripheral stalks and two a-subunits. Consistent with these results, analysis of the equilibrium *pmf* revealed an H^+^/ATP ratio that was doubled compared to that of the wild type. These results indicate that the coupling stoichiometry of the H^+^ ions is proportional to the number of a-subunits. The additivity in H^+^ stoichiometry means that each a-subunit tightly couples H^+^ translocation and rotation of the c-ring, regardless of the presence of other a-subunits. This is consistent with the half-channel model, which assumes that the probability of H^+^ translocation between the a and the c-subunits depends primarily on the relative position of these subunits, which explains well the functional independence of the a-subunits. Based on these considerations, we propose that the number of H^+^ ions transported coupled with rotation is determined not only by the number of c-subunits constituting the c-ring but also by the number of a-subunits as follows:

6 $${N}_{{H}^{+}/{turn}}={N}_{c}\times {N}_{a}$$

where ${N}_{{H}^{+}/{turn}}$, ${N}_{c}$, and ${N}_{a}$ represent the total number of H^+^ ions per turn, the number of c*-*subunit in the c*-*ring, and the number of a*-*subunit in F_o_, respectively. However, one might point out the inconsistency between the biochemical results and the structural analysis with cryo-EM. The proportion of molecules with three peripheral stalks was extremely low, clearly lower than the average number suggested by the biochemical results. This is attributable to the dissociation of the peripheral stalks during sample preparation for cryo-electron microscopy. To confirm this point, it is necessary to develop multi-stalk F_o_F_1_ in which peripheral stalks stably bind to F_1_ and to more accurately analyze the correlation between the number of the a*-*subunit and the H^+^ stoichiometry.

As mentioned above, the increased H^+^/ATP ratio suggests the functional independence of the a*-*subunits. However, this does not guarantee kinetic independence among the a-subunits. Thus, an arising question is: ‘Can F_o_ with multiple a*-*subunits rotate the c-ring at the same rate as the wild-type F_o_?’. In other words, ‘Do the a-subunits conduct H^+^ translocation without mutual interference?’ This is a reasonable question considering that, before each 36° rotation step of the c*-*ring, all a-subunits must complete the H^+^ translocation with the interacting c-subunits. Therefore, the time constant for each 36° rotation step is expected to be proportional to the number of a-subunits, meaning that the rate constant would decrease inversely.

Our biochemical experiments suggest that ATP hydrolysis-coupled proton pump activity was lower than that of the wild type (Supplementary Table 1). This aligns with the above expectation. However, other scenarios are possible: the engineered F_o_F_1_ may be more susceptible to the *pmf* progressively generated upon proton transport, and the engineered F_o_F_1_ may affect the catalytic activity of F_1_. Therefore, a quantitative and systematic analysis is necessary to verify this issue.

Regarding the lower activity of the mutant, a naive question could arise: ‘Why can the mutant still carry out the ATP synthesis reaction under lower *pmf* conditions despite its lower activity?’. The reason is as follows: the *pmf* required for F_o_F_1_ to synthesize ATP is determined by the *pmf*_eq_ at which the synthesis and hydrolysis reaction rates are balanced. The present study shows that the engineered F_o_F_1_ with multiple a-subunits doubles the H^+^/ATP ratio, resulting in a halved *pmf*_eq_. Therefore, although the engineered ATP synthase has lower catalytic activity, the mutant enzyme can continue the ATP synthesis reaction under low *pmf* conditions where the wild-type enzyme is unable to synthesize ATP.

This study has shown that, in principle, ATP synthase has the capacity to expand its H^+^/ATP ratio to more than double. To date, experimentally confirmed H^+^/ATP ratios of F_o_F_1_ have only been ~3–4. Even structural estimates suggest that 5.0 is the maximum for photosynthetic bacteria. Regarding this point, an N-type ATPase—considered a subtype of the F-type—has been reported to have an exceptionally large c-ring composed of 17 c-subunits^29^. Although the function and overall structure of this enzyme remain unknown, its predicted H^+^/ATP ratio is 5.7. The present study demonstrates that the H^+^/ATP ratio can be significantly increased by genetically engineering ATP synthase to increase the number of a-subunits, without resorting to such a large c-ring. Such genetic mutations may have arisen over the course of evolution. Indeed, we found that the gene operon of ATP synthase from *Acidaminococcus fermentans* shows the gene fusion of the δ and the α subunits (Supplementary Fig. 9). In addition, the N-terminal domain of δ is missing. These features are well consistent with the δ_ΔN_-α fused F_o_F_1_ we designed. Thus, it is highly likely that *A. fermentans* F_o_F_1_ has a multi-stalk structure and can synthesize ATP under low *pmf* conditions. We also found that other species show similar features (UniProt ID: G4Q3K6, A0A1I2C5T3), suggesting more possibility of a multi-stalk F_o_F_1_ in nature. Thus, the present study suggests the unexpected diversity in the design principles of the F_o_F_1_ ATP synthase, which awaits further experimental verification.

From an engineering standpoint, the findings of the present study may provide future directions for cell engineering. So far, in microbial fermentation, attention has primarily been devoted to developing metabolic pathways and altering metabolic fluxes, while little effort has been made to optimize the intracellular concentrations and ratios of NAD(P)H and ATP, which are fundamental to cellular bioenergetics. When ATP synthesis is driven by oxidative phosphorylation, the amount of ATP produced depends on the extent of NADH oxidation. In such cases, an increase in the H^+^/ATP ratio translates into a higher NADH/ATP ratio. This could have a significant impact on microbial fermentations. In particular, it could enhance bioproduction in photosynthetic bacteria when ATP is the bottleneck factor^22,30^. To verify this possibility, it will be necessary to investigate how introducing ATP synthase with a modified H^+^/ATP ratio affects cell growth and product formation. Additionally, such insights are likely to shed light on the physiological role of naturally occurring multi-stalk F_o_F_1_, which may also exist in nature.

## Methods

### Preparation of F_o_F_1_

In this study, *Bacillus* PS3 F_o_F_1_-ε_ΔC_, which has a 10× His-tag at the N-terminus of the β subunit and lacks the inhibitory C-terminal domain of the ε subunit^17,24^ was used as the wild-type. The engineered δ_ΔN_-α fusion construct of *Bacillus* PS3 F_o_F_1_-ε_ΔC_ lacks the full-length δ subunit and has a δ_ΔN_-α fused subunit in which the N-terminal domain (residues 2–104) of the δ subunit is deleted and its C-terminus is fused to the N-terminus (without Met) of the α subunit via a short linker. The wild-type and engineered F_o_F_1_s were expressed in *E. coli* DK8 cells, which lack endogenous F_o_F_1_ genes, by incubating in Super broth at 37 °C for 20 h. Cultured cells were suspended in a solution (10 mM HEPES, pH 7.5, 5 mM MgCl_2_, and 10% (v/v) glycerol) and disrupted by sonication. After removing the cell debris at 9100 × *g* for 45 min, membrane fraction was collected by centrifugation for 131,500 × *g* for 1 h at 4 °C. F_o_F_1_ was solubilized from the membrane fraction by adding 0.5% (w/v) LMNG (NG310; Anatrace, USA) and incubating for 30 min at 30°C. After centrifugation at 162,000 × *g* for 30 min, the solubilized fraction was applied to a Ni-Sepharose column pre-equilibrated with M buffer (20 mM potassium phosphate buffer and 100 mM KCl, pH 7.5) containing 0.005% LMNG. The column was washed with M buffer containing 20 mM imidazole and 0.005% LMNG, and F_o_F_1_ was eluted with M buffer containing 200 mM imidazole and 0.005% LMNG. The eluted F_o_F_1_ fractions were concentrated before being applied to a Superdex 200 Increase 10/300 column (Cytiva) equilibrated with gel filtration buffer (20 mM HEPES, pH7.5, 100 mM NaCl, and 0.005% LMNG). The peak fractions corresponding to F_o_F_1_ were collected and concentrated to 5–10 mg/mL, frozen with liquid nitrogen, and stored at −80 °C until use. The protein concentrations were determined using a BCA protein assay kit (Pierce) with bovine serum albumin as a standard. The molecular weight of the protein was calculated based on the sequence and subunit stoichiometry. For the δ_ΔN_-α fused F_o_F_1_, the molecular weight was calculated assuming an average of two peripheral stalks per F_o_F_1_ molecule.

### Measurement of ATP synthesis/hydrolysis activity of F_o_F_1_

ATP synthesis/hydrolysis activity of F_o_F_1_ was measured using a luciferin-luciferase system at 25 °C, as described previously^17^. F_o_F_1_-reconstituted PLs were prepared as described^17^. Then, 300 µL of the PLs were mixed with 700 µL of acidic buffer containing 50 mM MES or HEPES buffer, 0.143–14.3 mM NaH_2_PO_4_, 6.7 mM KCl, 49 mM NaCl, 4 mM MgCl_2_, and 600 mM sucrose and NaOH to obtain the desired pH, and then ADP and valinomycin were added to a final concentration of 20-640 µM and 200 nM, respectively. After incubation for 10–24 h at 25 °C for acidification, base assay medium was prepared by mixing 25 µL of the luciferin/luciferase mixture (2× concentration of CLSII solution in ATP bioluminescence assay kit, Roche, and 5 mM luciferin), 800 µL of the base buffer (380 mM HEPES buffer, 0.1125–11.25 mM NaH_2_PO_4_, 5.63 mM KCl, 55 mM NaCl, 4 mM MgCl_2_, KOH to adjust K^+^ concentration and NaOH to adjust pH), 50-100 µL of ATP and ADP to obtain the desired concentration, and water to adjust the total volume to 900 µL and incubated for 10 min for equilibrium. Then, the 100 µL of acidified PLs was injected into the base assay medium to initiate the ATP synthesis reaction, and the ATP synthesis/hydrolysis activity was monitored with the luciferin/luciferase assay system using a luminometer (Luminescencer AB2200, ATTO). For calibrating luminescence light intensity to ATP concentration, 10 μL of 10 μM ATP was added. The rate was determined from the initial slope of the linear regression of the time courses. The ΔpH was obtained by subtracting pH_in_ from pH_out_, which was determined by directly measuring the pH using a glass electrode. Transmembrane electrical potential, Δψ, was estimated from the Nernst equation.

### Other assays

SDS-PAGE analysis was performed with 10–20% (w/v) gradient gels. The gels were stained with Coomassie Brilliant Blue (CBB Stain One Super, Nacalai Tesque, Japan) and imaged with a ChemiDoc Imaging System (BIORAD, USA). The band intensity of each subunit was measured using the gel analyzer tool of Fiji (ImageJ 1.54 f) software. ATPase activity measurements of PLs using an ATP regeneration system were performed at 25 °C in the ATPase assay solution (50 mM HEPES-KOH, pH 7.5, 100 mM KCl, 5 mM MgCl_2_, 2 mM ATP, 1 μg/ml Carbonyl cyanide-p-trifluoromethoxyphenylhydrazone, 2.5 mM phosphoenolpyruvate, 100 µg/mL lactate dehydrogenase, 100 µg/mL pyruvate kinase and 0.2 mM NADH) as described previously^31^. ATP-driven H^+^-pumping activity was measured by quenching of ACMA (9-amino-6-chloro-2-methoxyacridine) fluorescence at 25 °C in PA4 buffer (10 mM HEPES-KOH, pH 7.5, 100 mM KCl, 5 mM MgCl_2_) supplemented with 0.3 μg/ml ACMA and 1.0 μg/ml F_o_F_1_-reconstituted PLs^31^.

### Cryo-EM grid preparation and data collection

After adding 0.05% lysophosphatidylcholine (1-palmitoyl-2-hydroxy-sn-glycero-3-phosphocholine), 3.0 µL of purified protein (3–6 mg/mL) was loaded onto the glow-discharged Quantifoil R1.2/1.3 grids using a Vitrobot Mark IV (Thermo Fisher Scientific). Grids were blotted for 5 s with a blotting force of 15 under 100% humidity at 18 °C, and flash-frozen in liquid ethane. Data were collected using a 300 kV Titan Krios electron microscope (Thermo Fisher Scientific) with a Falcon 4i direct detector device camera with Selectris-X automated with EPU software. Images were recorded in electron counting mode by recording 50 movie frames with an exposure rate of 1.0 e^−^/Å^2^ per frame. The defocus range was 0.8–2.0 μm, and the original pixel size was 0.75 Å.

### Cryo-EM data processing

All image processing steps were performed using cryoSPARC v4.3.0. Details of the image processing workflow are described in Supplementary Fig. 4. A total of 56,081 micrographs were first motion corrected, and the CTF was estimated by patch CTF estimation. Particles were manually picked, and templates for particle selection were generated from 2D classification. After template picking, the selected 4,316,627 particles were subjected to 2D classification. Then, further selections with ab initio 3D reconstruction, heterogeneous, homogeneous, and non-uniform refinements were performed. After non-uniform refinement, all particles were sequentially subjected to a focused 3D refinement using masks for each of the three peripheral stalks, including the N-terminal region of the α subunit, the C-terminal region of the δ subunit and the hydrophilic region of the b_2_-subunits. Each mask was generated from three rotational states of wild-type *Bacillus* PS3 F_o_F_1_ (PDBs 6N2Z, 6N30, and 6N2Y). Eight classes, including one to three peripheral stalks and three rotational states of F_o_F_1_, were identified. The three datasets were collected, merged and refined with non-uniform refinement, resulting in an overall resolution of 2.5–3.2 Å. Further refinement with the F_o_ mask resulted in a map of the F_o_ region with an improved resolution of 3.5–6.6 Å. The resolution was estimated using the FSC criterion of 0.143 threshold. The cryo-EM data collection and refinement statistics are shown in Supplementary Tables 2 and 3. The local resolution maps, FSC curves, orientation distribution plots and Model-to-map fits are shown in Supplementary Figs. 10–12.

### Model building

Models were built and refined in COOT v0.9.8.93 and PHENIX v1.20.1-4487. using PDB 6N2Z, 6N30, and 6N2Y as the initial model. Validation statistics are shown in Supplementary Tables 2 and 3. A composite map was generated by combining the F_1_ region of the triple-stalk F_o_F_1_ map and the F_o_ map obtained through local refinement with the F_o_ mask, using UCSF ChimeraX v1.8 for illustration purposes only. Figures were prepared using PyMOL v2.4.0&2.5.2, UCSF Chimera v1.17.3, and UCSF ChimeraX v1.5&1.8. RMSD values for Cα-atoms were calculated using PyMOL 2.4.0 align command without outlier rejection.

### Reporting summary

Further information on research design is available in the Nature Portfolio Reporting Summary linked to this article.

## Supplementary information

Supplementary Information

Reporting Summary

Transparent Peer Review file

## Source data

Source Data file

## Data Availability

The cryo-EM maps and models generated in this study were deposited to EMDB and PDB under the following accession codes: EMD-61339; EMD-61340; EMD-61341; EMD-61342; EMD-61343; EMD-61344; EMD-61345; EMD-61346; EMD-61347; EMD-61348; EMD-61349; EMD-61350; EMD-61351; EMD-61352; EMD-61353; EMD-61354, and 9JC1; 9JC2. Previously published structures used in this study are also available from PDB under the following accession codes: 6N2Z; 6N30, and 6N2Y. Source data are provided with this paper.
