# Supplementary material for: Engineering of ATP synthase for enhancement of proton-to-ATP ratio
Source: Nat Commun. 2025 Jul 3;16:5410. doi: 10.1038/s41467-025-61227-w (PMC12229609; doi:10.1038/s41467-025-61227-w)
Supplement: Supplementary file 1 — Supplementary Information [file 41467_2025_61227_MOESM1_ESM.pdf]

SUPPLEMENTARY INFORMATION

Engineering of ATP synthase for enhancement of proton-to-ATP ratio

Hiroshi Ueno[1*], Kiyoto Yasuda[1], Norie Hamaguchi-Suzuki[2,3], Riku Marui[1], Naruhiko Adachi[4,5], Toshiya Senda[4], Takeshi Murata[2], Hiroyuki Noji[1*]

[1]Department of Applied Chemistry, Graduate School of Engineering, The University of Tokyo, Tokyo, Japan, [2]Department of Chemistry, Graduate School of Science, Chiba University, Chiba, Japan, [3]Department of Pharmacology, Graduate School of Medicine, Chiba University, Chiba, Japan, [4]Structural Biology Research Center, Institute of Materials Structure Science, High Energy Accelerator Research Organization (KEK), Ibaraki, Japan. [5]Life Science Center for Survival Dynamics, Tsukuba Advanced Research Alliance (TARA), University of Tsukuba, Ibaraki, Japan.

[*]Corresponding authors: hueno@g.ecc.u-tokyo.ac.jp and hnoji@g.ecc.u-tokyo.ac.jp

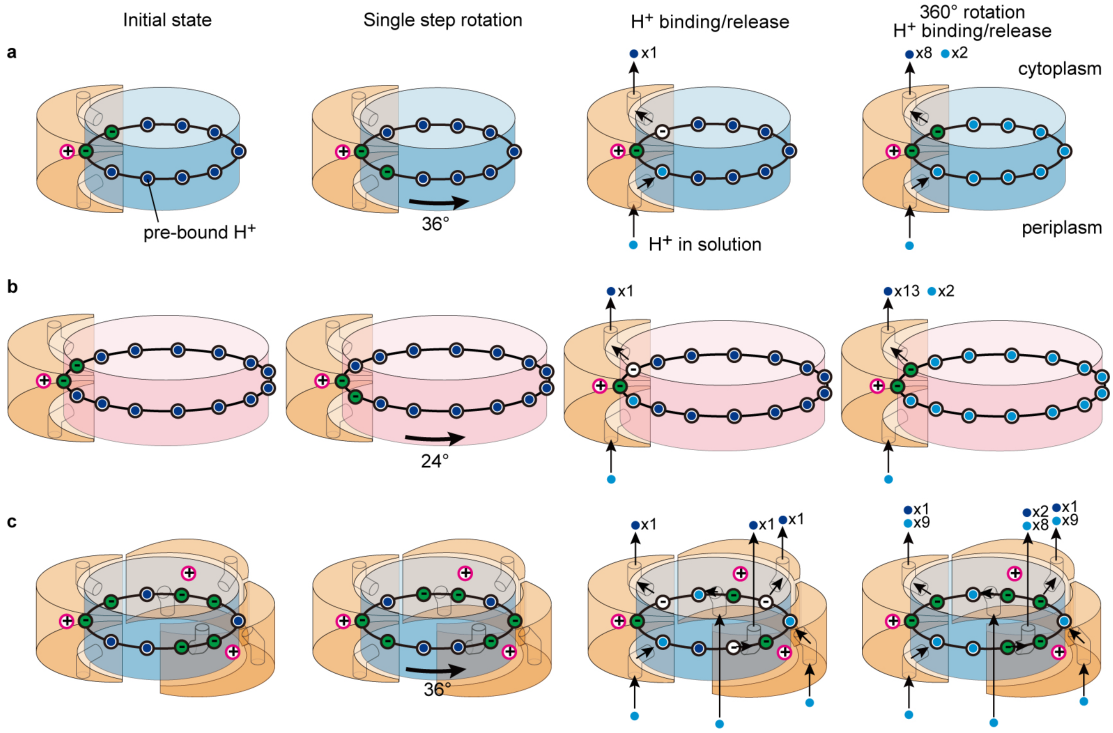

Supplementary Figure 1. Models of H^+ translocation through F_o per one revolution. The highly conserved arginine residues of the a-subunit (orange) are depicted with pink open circles. The c-subunits are depicted as black open circles with pre-bound H^+ at the initial state (deep blue circles) and H^+ incorporated from the solution (light blue circles) and, for clarity, deprotonated c-subunits facing the a-subunit at the initial state are depicted as green circles. **a**, Model with c_{10}-ring and single a-subunit. At the initial state, two c-subunits are deprotonated. After single-step rotation, pre-bound H^+ is released into the cytoplasmic half-channel due to its interaction with the positively charged arginine residue of the a-subunit, and the H^+ in the periplasm enters through the periplasmic half-channel and is transferred to the deprotonated c-subunit. In this process, one H^+ is transferred from the periplasm to the cytoplasm and a total of 10 H^+s are transferred in one revolution. **b**, Model with c_{15}-ring and a single a-subunit. A total of 15 H^+s are transferred in one revolution. **c**, Model with a c_{10}-ring and triple a-subunits. A total of 30 H^+s are transferred in one revolution.

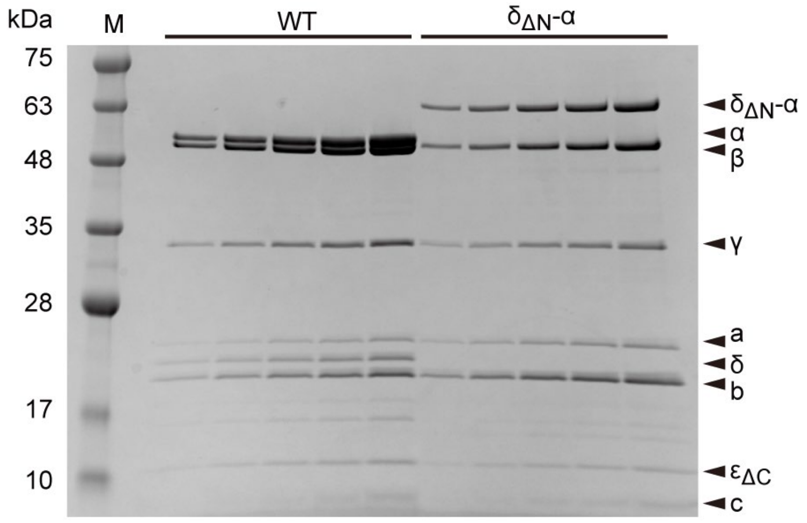

Supplementary Figure 2. SDS-PAGE of wild-type F_oF_1 and $\delta_{\Delta N}$-α fused F_oF_1. Lanes were loaded with 0.53, 0.88, 1.32, 1.76, 2.64 μg of the purified wild-type F_oF_1, and 0.50, 0.84, 1.26, 1.68, 2.52 μg of the purified $\delta_{\Delta N}$-α fused F_oF_1.

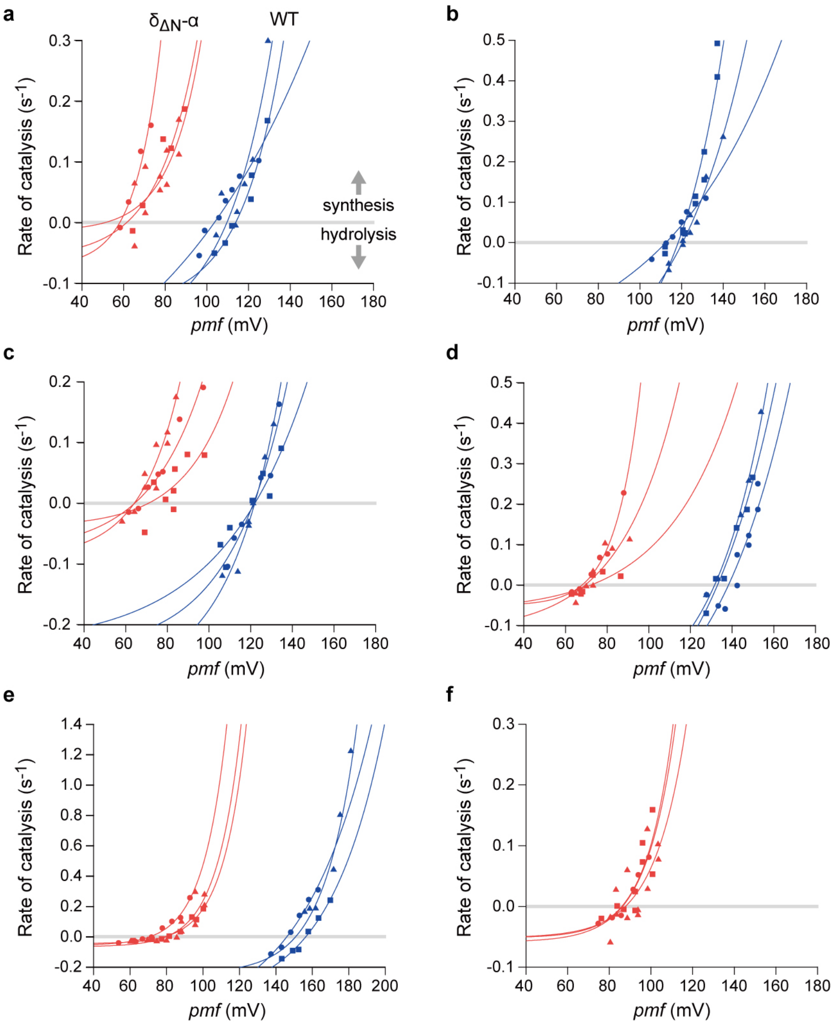

Supplementary Figure 3. Comparison of the *pmf* dependence of the rate of catalysis at different Q conditions. The results of three independent experiments from different batches of PLs (circles triangles, and squares) at each Q condition are shown. The plots for the $\delta_{\Delta N}$-α fused F_oF_1 (red) and wild-type F_oF_1 (blue) are shown. **a**, Q = 0.078; [ATP] = 500 nM, [ADP] = 640 μM, [Pi] = 10 mM. **b**, Q = 0.208; [ATP] = 500 nM, [ADP] = 240 μM, [Pi] = 10 mM. **c**, Q = 0.625; [ATP] = 500 nM, [ADP] = 80 μM, [Pi] = 10 mM. **d**, Q = 2.5; [ATP] = 500 nM, [ADP] = 20 μM, [Pi] = 10 mM. **e**, Q = 12.5; [ATP] = 500 nM, [ADP] = 40 μM, [Pi] = 1 mM. **f**, Q = 62.5; [ATP] = 500 nM, [ADP] = 80 μM, [Pi] = 0.1 mM.

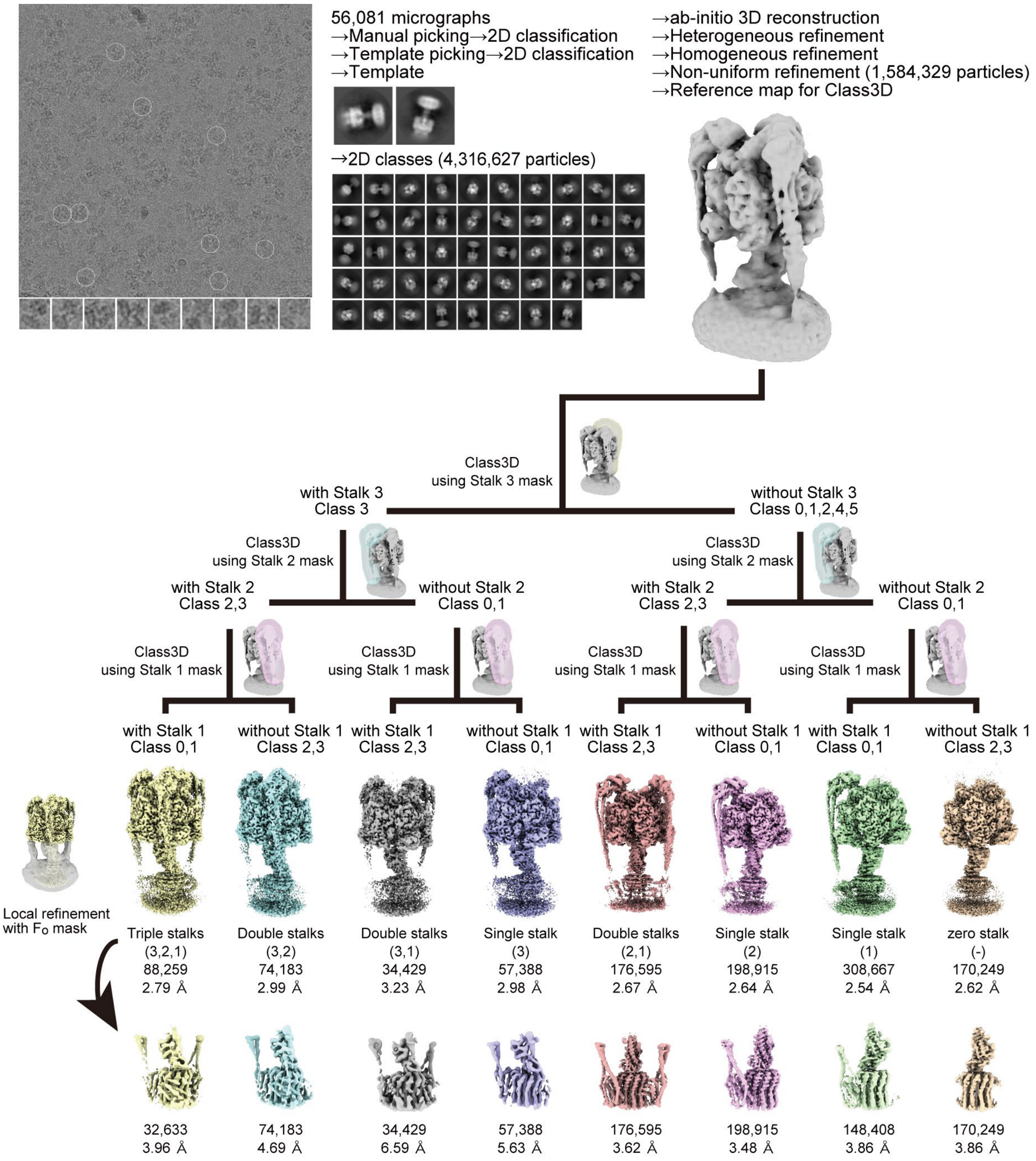

Supplementary Figure 4. Image processing workflow. Typical micrographs, 2D class averages and the image processing workflow are shown. The masks for Stalk 1, 2, and 3 were generated using the PDB models (6N2Z, 6N30, and 6N2Y), respectively. The number in parentheses indicates the position of each peripheral stalk as seen in each structure.

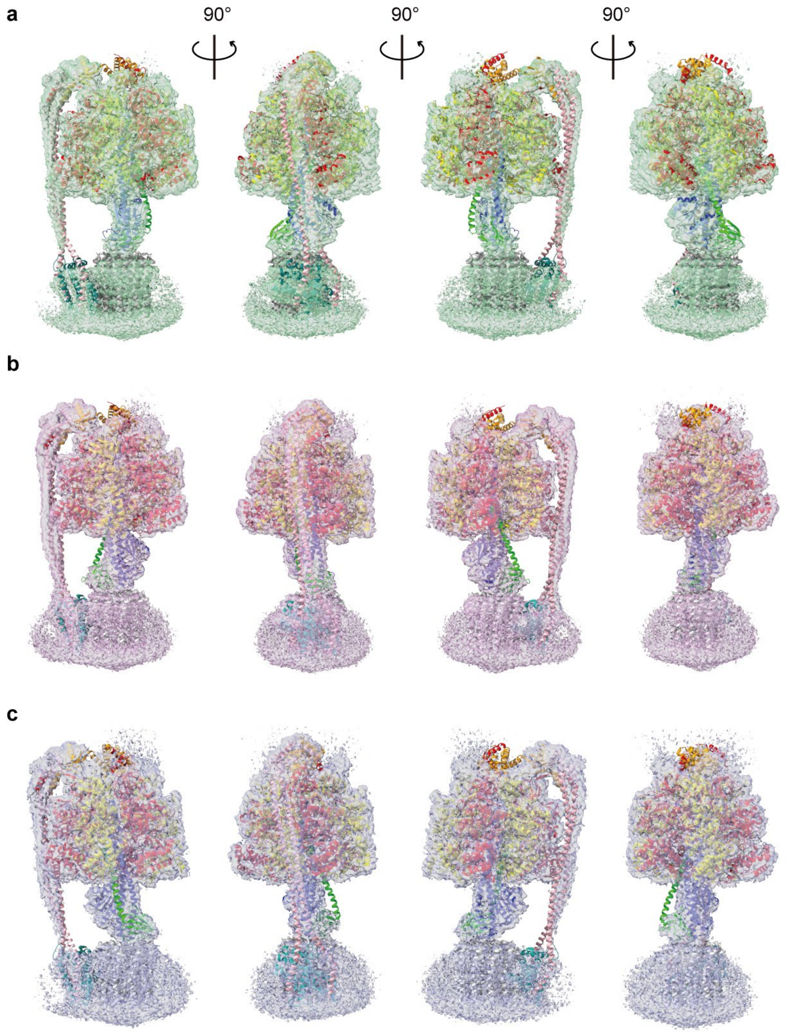

Supplementary Figure 5. Three rotational states of F_oF_1 with a single peripheral stalk. a, **b**, and **c**, The cryo-EM maps of the three single-stalk F_oF_1 with Stalk 1 (**a**), 2 (**b**), and 3 (**c**) were fitted with the structures of *Bacillus* PS3 F_oF_1 (PDBs 6N2Z, 6N30, and 6N2Y), respectively.

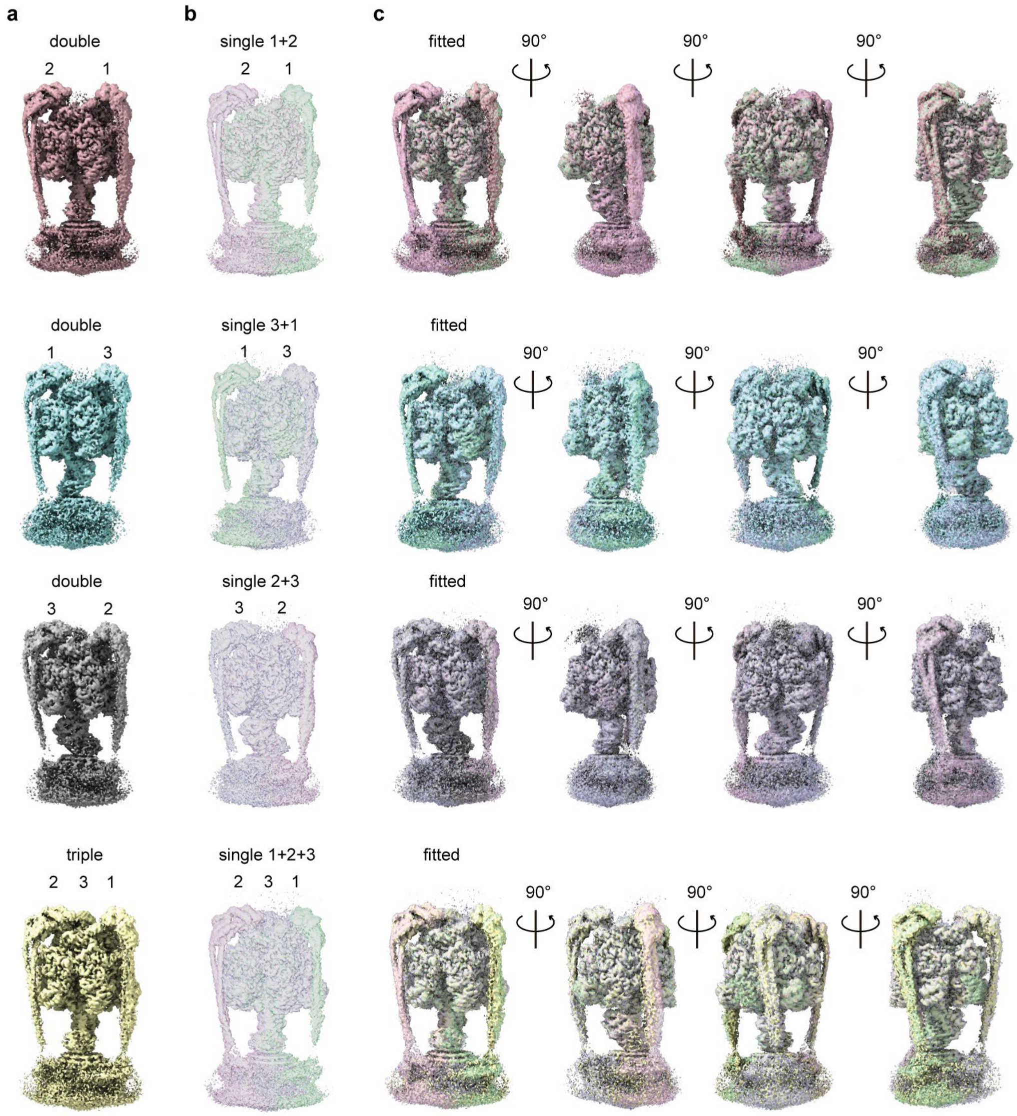

Supplementary Figure 6. Double- and triple-stalk F_oF_1 vs single-stalk F_oF_1. **a**, **b**, The maps of the double- and triple-stalk F_oF_1 (**a**) and the corresponding maps of single-stalk F_oF_1 (**b**). **c**, Overlay of the maps shown in (**a**) and (**b**), respectively.

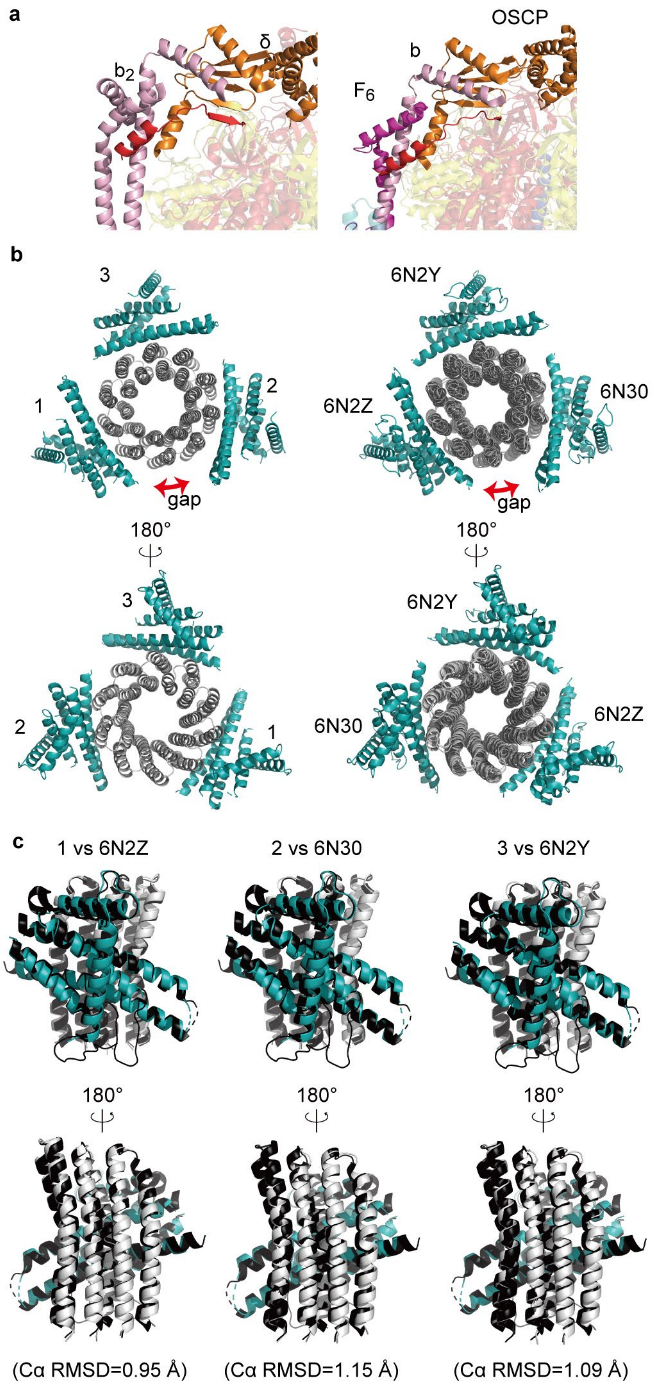

Supplementary Figure 7. Structural comparison between the triple-stalk F_oF_1 and the wild-type F_oF_1. a, The side view of the interaction site between δ (orange), α (red), and b_2 (pink) subunits of the wild-type *Bacillus* PS3 F_oF_1 (left, PDB: 6N2Y), and OSCP (orange), α (red), F6 (magenta), and b (pink) subunits of bovine F_oF_1 (right, PDB: 6YY0). **b**, F_o of the triple-stalk F_oF_1 (left) and the wild-type *Bacillus* PS3 F_oF_1 when the three rotational states of the wild-type *Bacillus* PS3 F_oF_1 (PDB: 6N2Z, 6N30, and 6N2Y) are aligned with the γ subunit (right). View from the F_o side. **c**, Superposition of each a_1c_3 unit in the triple-stalk F_oF_1 with that in the corresponding state of the wild-type *Bacillus* PS3 F_oF_1.

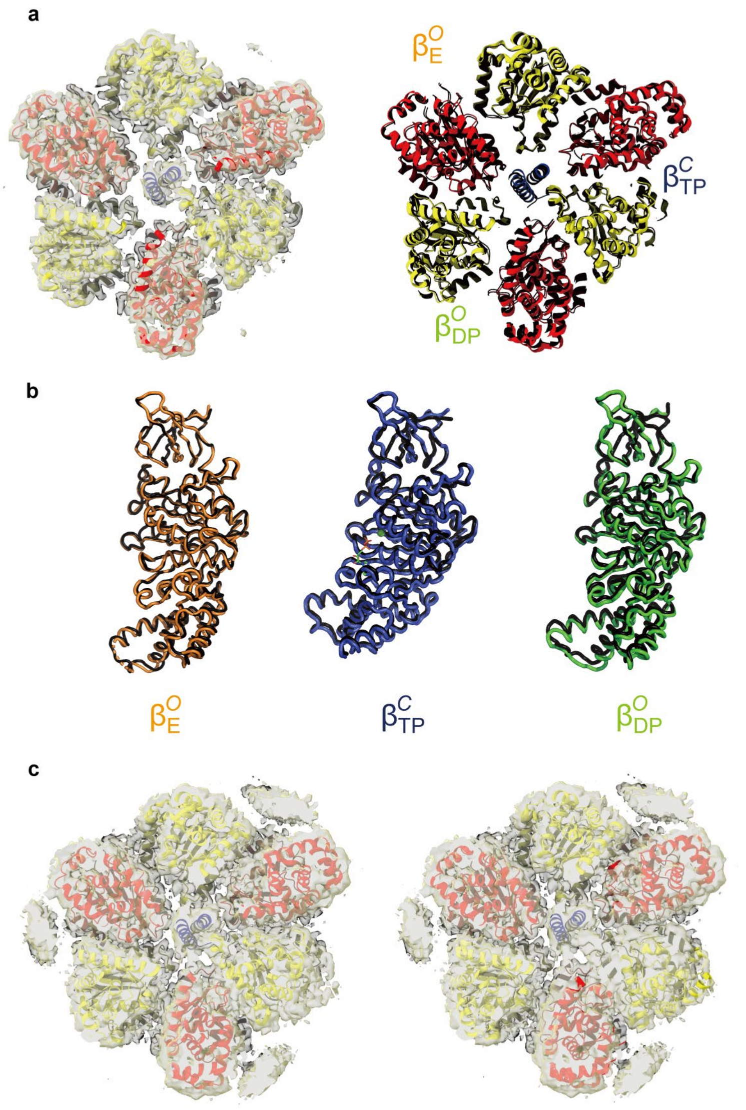

Supplementary Figure 8. F_1 part of the triple-stalk F_oF_1. a, The cryo-EM map and the atomic model of the F_1 part in the triple-stalk F_oF_1 viewed from the F_o side (left). The α, β, and γ subunits are colored in red, yellow, and blue, respectively. Superposition of the $\alpha_3\beta_3$-ring of the triple-stalk F_oF_1 (red and yellow) with that of the *Bacillus* PS3 F_oF_1-$\varepsilon_{\Delta C}$ under unisite catalysis conditions (black, PDB: 7XKP) (right). **b**, Each β subunit of the triple-stalk F_oF_1 (β_E^O, β_{TP}^C, and β_{DP}^O) and the *Bacillus* PS3 F_oF_1-$\varepsilon_{\Delta C}$ (β_E^{Open}, β_{TP}^{Close}, and β_{DP}^{Open}, PDB: 7XKP) were superimposed on the N-terminal β-barrel (β2-82). **c**, Superposition of the atomic models of the F_1 part in the triple-stalk F_oF_1 (left) and that in the nucleotide-depleted *Bacillus* PS3 F_oF_1-$\varepsilon_{\Delta C}$ (right) onto the cryo-EM map of the triple stalks F_oF_1 at lower density threshold than that shown in (**a**).

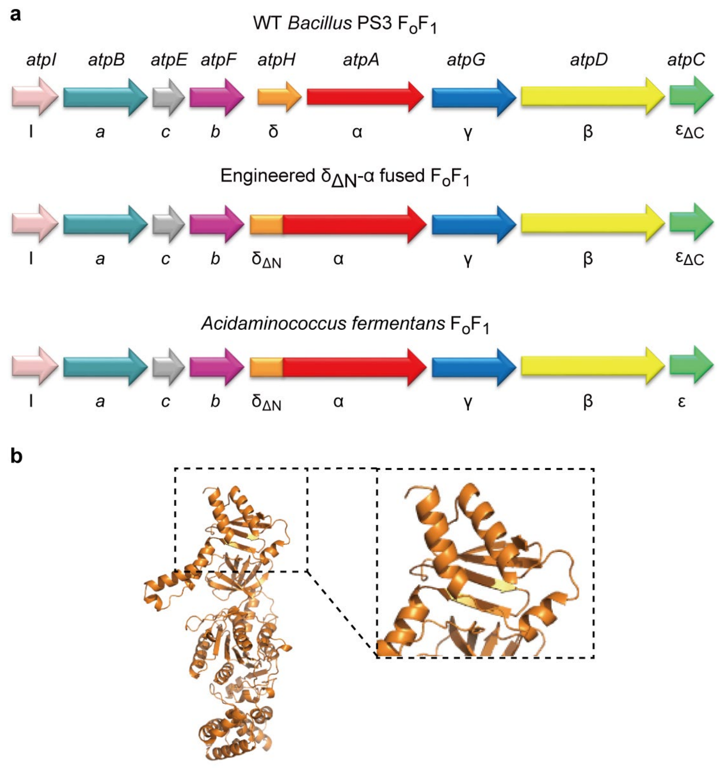

Supplementary Figure 9. The *atp* operon of *Acidaminococcus fermentans*. a, Gene order of the wild-type *Bacillus* PS3 F_oF_1 and the engineered $δ_{ΔN}$-α fused F_oF_1 used in this study, and the F_oF_1 from *Acidaminococcus fermentans*. **b,** The predicted structure of the $δ_{ΔN}$-α fused subunit of F_oF_1 from *Acidaminococcus fermentans* in the AlphaFold Protein Structure Database (UniProt ID: D2RLW6). The structure is also similar to the corresponding part of the engineered $δ_{ΔN}$-α fused F_oF_1.

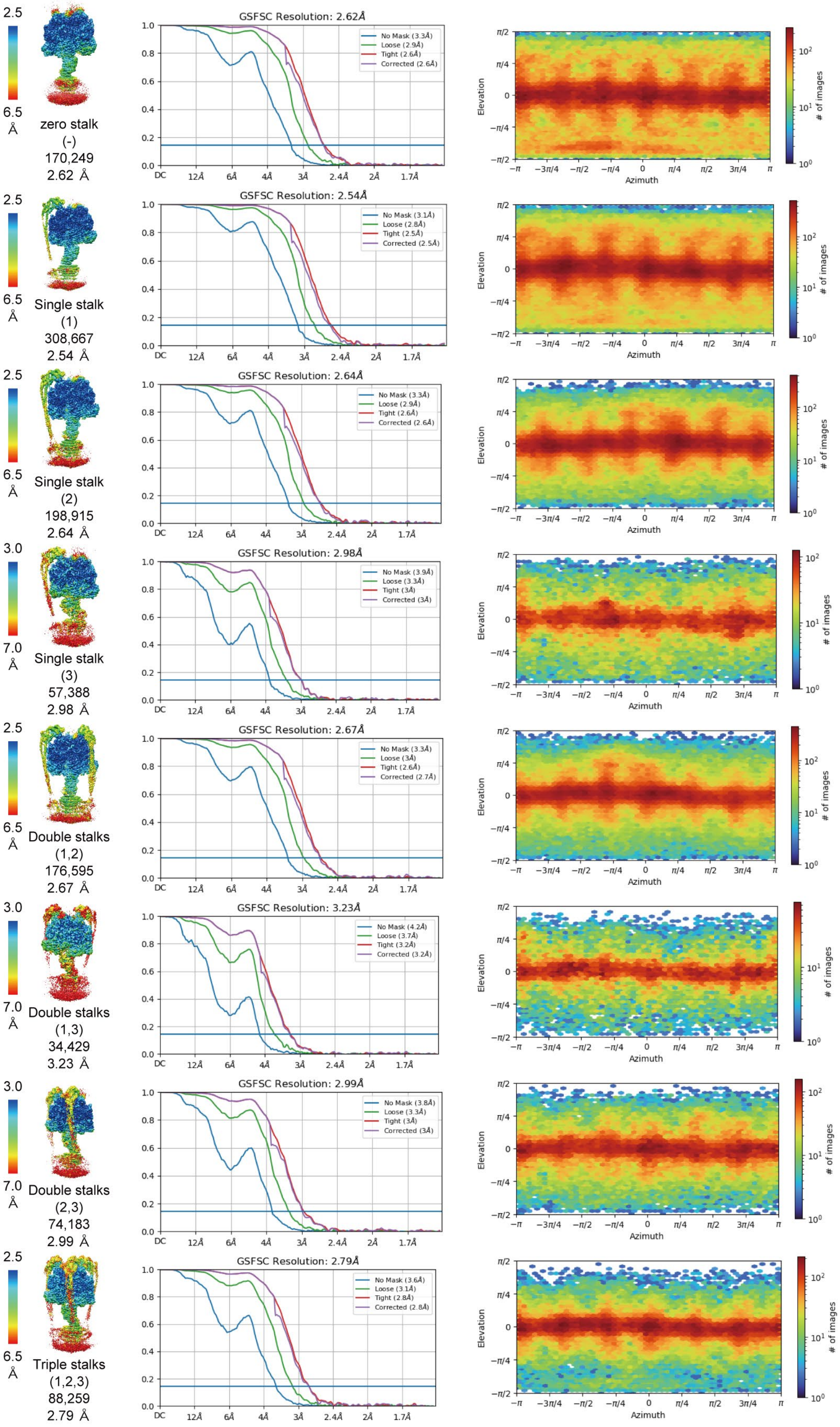

Supplementary Figure 10. Local resolution maps, FSC curves, and orientation distribution plots for F_oF_1 structures. For each F_oF_1 cryo-EM reconstruction, local resolution maps, Gold standard Fourier shell correlation (GSFSC) curves, and orientation distribution plots are shown.

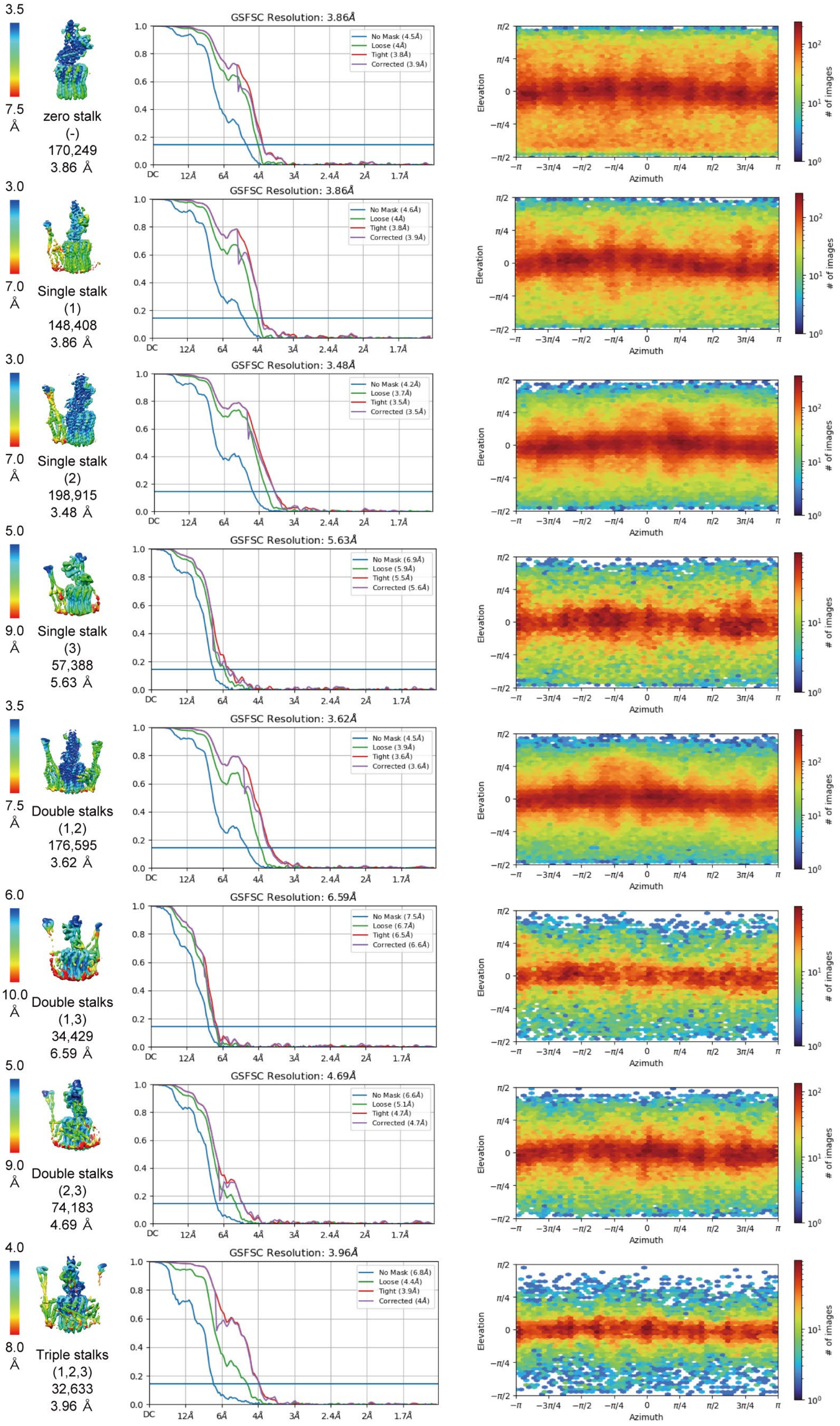

Supplementary Figure 11. Local resolution maps, FSC curves, and orientation distribution plots for F_o structures. For each F_o cryo-EM reconstruction, local resolution maps, Gold standard Fourier shell correlation (GSFSC) curves, and orientation distribution plots are shown.

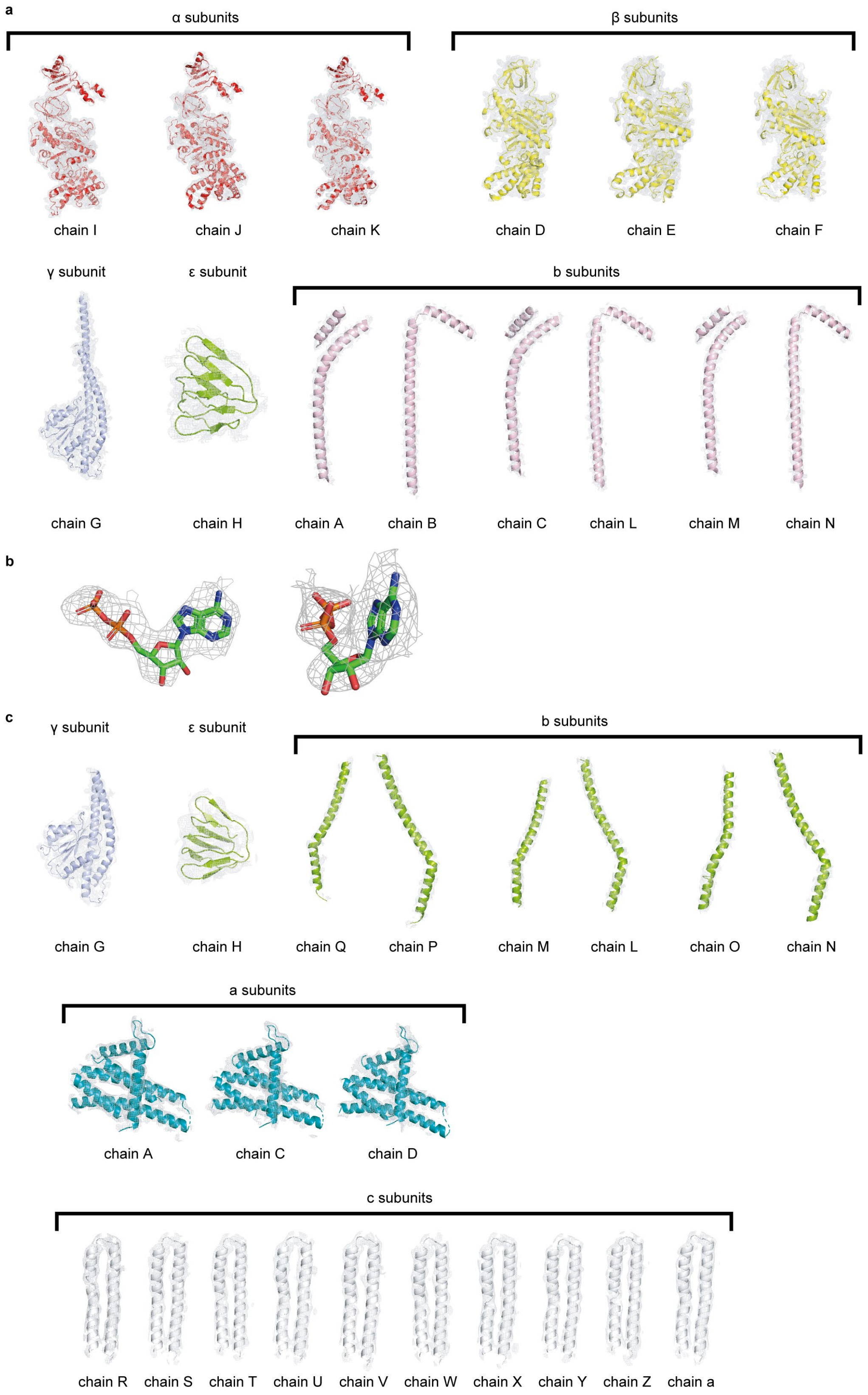

Supplementary Figure 12. Model-to-map fits. a, Model-to-map fits for each subunit in the F_1 region (PDB ID: 9JC1). **b,** Bound ADP in the β subunit of F_1 shown with stick model and map from two different viewing angles. **c,** Model-to-map fits for each subunit in the F_o region (PDB ID: 9JC2).

Supplementary table 1. H^+-pumping and ATPase activity of F_oF_1 reconstituted proteoliposomes at 2 mM ATP. The results of *n* repeated experiments from two different batches of proteoliposomes using the same batch of enzymes are shown.

Protein	H^+-pumping activity (n = 6)	ATPase activity (n = 8)
Wild-type F_oF_1	77 ± 4% ACMA quench	53 ± 6 s^{-1}
$\delta_{\Delta N}$-α fused F_oF_1	18 ± 5% ACMA quench	21 ± 3 s^{-1}

Supplementary table 2. Cryo-EM data collection, refinement and validation statistics for F_oF_1 with 0, 1, and 2 peripheral stalks.

	#1 Zero-stalk F_oF_1 and F_o (-) (EMD-61347) (EMD-61354)	#2 Single - stalk F_oF_1 and F_o (1) (EMD-61341) (EMD-61348)	#3 Single-stalk F_oF_1 and F_o (2) (EMD-61342) (EMD-61349)	#4 Single-stalk F_oF_1 and F_o (3) (EMD-61343) (EMD-61350)	#5 Double-stalk F_oF_1 and F_o (1, 2) (EMD-61344) (EMD-61351)	#6 Double-stalk F_oF_1 and F_o (1, 3) (EMD-61345) (EMD-61352)	#7 Double-stalk F_oF_1 and F_o (2, 3) (EMD-61346) (EMD-61353)
**Data collection and processing**							
Magnification	165,000	165,000	165,000	165,000	165,000	165,000	165,000
Voltage (kV)	300	300	300	300	300	300	300
Electron exposure (e–/Å^2)	50	50	50	50	50	50	50
Defocus range (µm)	-0.8 to -2.0	-0.8 to -2.0	-0.8 to -2.0	-0.8 to -2.0	-0.8 to -2.0	-0.8 to -2.0	-0.8 to -2.0
Pixel size (Å)	0.75	0.75	0.75	0.75	0.75	0.75	0.75
Symmetry imposed	C1	C1	C1	C1	C1	C1	C1
Initial particle images (no.)	4,316,627	4,316,627	4,316,627	4,316,627	4,316,627	4,316,627	4,316,627
Final particle images (no.)	170,249	308,667 (F_oF_1) 148,408 (F_o)	198,915	57,388	176,595	34,429	74,183
Map resolution (Å)	F_oF_1 2.62 F_o 3.86	F_oF_1 2.54 F_o 3.86	F_oF_1 2.64 F_o 3.48	F_oF_1 2.98 F_o 5.63	F_oF_1 2.67 F_o 3.62	F_oF_1 3.23 F_o 6.59	F_oF_1 2.99 F_o 4.69
FSC threshold	0.143	0.143	0.143	0.143	0.143	0.143	0.143

Supplementary table 3. Cryo-EM data collection, refinement and validation statistics for F_oF_1 with triple peripheral stalks.

	#8 Triple-Stalk F_oF_1 (EMD-61339)	#9 F_1 of Triple-stalk F_oF_1 (EMD-61339) (PDB 9JC1)	#10 F_o of Triple-stalk F_oF_1 (EMD-61340) (PDB 9JC2)
**Data collection and processing**			
Magnification	165,000	165,000	165,000
Voltage (kV)	300	300	300
Electron exposure (e–/Å^2)	50	50	50
Defocus range (μm)	-0.8 to -2.0	-0.8 to -2.0	-0.8 to -2.0
Pixel size (Å)	0.75	0.75	0.75
Symmetry imposed	C1	C1	C1
Initial particle images (no.)	4,316,627	4,316,627	4,316,627
Final particle images (no.)	88,259	88,259	32,633
Map resolution (Å)	2.79	2.79	3.96
FSC threshold	0.143	0.143	0.143
**Refinement**			
Initial model used (PDB code)		6N2Z	6N2Z
Model resolution (Å)		3.1	4.3
FSC threshold		0.5	0.5
Map sharpening *B* factor (Å^2)		49.8	96.1
Model composition			
Non-hydrogen atoms		28164	10826
Protein residues		3905	1875
Ligands		4MG,1ADP	N/A
B factors (Å^2)			
Protein		74.94	193.72
Ligand		65.44	N/A
R.m.s. deviations			
Bond lengths (Å)		0.004	0.003
Bond angles (°)		0.567	0.611
Validation			
MolProbity score		1.67	1.51
Clashscore		4.90	9.79
Poor rotamers (%)		2.28	0.56
Ramachandran plot			
Favored (%)		97.10	98.28
Allowed (%)		2.85	1.72
Disallowed (%)		0.05	0.00
