# Supplementary material for: Engineering of ATP synthase for enhancement of proton-to-ATP ratio
Source: Nat Commun. 2025 Jul 3;16:5410. doi: 10.1038/s41467-025-61227-w (PMC12229609; doi:10.1038/s41467-025-61227-w)
Supplement: Supplementary file 2 — Reporting Summary [file 41467_2025_61227_MOESM2_ESM.pdf]

nature portfolio

Corresponding author(s): Hiroshi Ueno, Hiroyuki Noji

Last updated by author(s): May 20, 2025

Reporting Summary

Nature Portfolio wishes to improve the reproducibility of the work that we publish. This form provides structure for consistency and transparency in reporting. For further information on Nature Portfolio policies, see our Editorial Policies and the Editorial Policy Checklist.

Statistics

For all statistical analyses, confirm that the following items are present in the figure legend, table legend, main text, or Methods section.

n/a	Confirmed	
☐	☒	The exact sample size (n) for each experimental group/condition, given as a discrete number and unit of measurement
☐	☒	A statement on whether measurements were taken from distinct samples or whether the same sample was measured repeatedly
☒	☐	The statistical test(s) used AND whether they are one- or two-sided<br>*Only common tests should be described solely by name; describe more complex techniques in the Methods section.*
☒	☐	A description of all covariates tested
☒	☐	A description of any assumptions or corrections, such as tests of normality and adjustment for multiple comparisons
☐	☒	A full description of the statistical parameters including central tendency (e.g. means) or other basic estimates (e.g. regression coefficient) AND variation (e.g. standard deviation) or associated estimates of uncertainty (e.g. confidence intervals)
☒	☐	For null hypothesis testing, the test statistic (e.g. F, t, r) with confidence intervals, effect sizes, degrees of freedom and P value noted<br>*Give P values as exact values whenever suitable.*
☒	☐	For Bayesian analysis, information on the choice of priors and Markov chain Monte Carlo settings
☒	☐	For hierarchical and complex designs, identification of the appropriate level for tests and full reporting of outcomes
☒	☐	Estimates of effect sizes (e.g. Cohen's d, Pearson's r), indicating how they were calculated

Our web collection on statistics for biologists contains articles on many of the points above.

Software and code

Policy information about availability of computer code

Data collection	EPU v3.1.0.4506REL
Data analysis	cryoSPARC v4.3.0, RELION v4.0.1, COOT v0.9.8.93, PHENIX v1.20.1-4487, UCSF Chimera v1.17.3, ChimeraX v1.5&1.8, PyMOL v2.4.0&2.5.2, ImageJ v1.54f, Igor Pro v8.03

For manuscripts utilizing custom algorithms or software that are central to the research but not yet described in published literature, software must be made available to editors and reviewers. We strongly encourage code deposition in a community repository (e.g. GitHub). See the Nature Portfolio guidelines for submitting code & software for further information.

Data

Policy information about availability of data

All manuscripts must include a data availability statement. This statement should provide the following information, where applicable:

- Accession codes, unique identifiers, or web links for publicly available datasets
- A description of any restrictions on data availability
- For clinical datasets or third party data, please ensure that the statement adheres to our policy

Cryo-EM maps and models generated in this study are available from the EMDB (accession numbers: EMD-61339 to 61354) and the PDB (PDB ID: 9JC1 and 9JC2). Source data are provided with this paper as a Source Data file.

Research involving human participants, their data, or biological material

Policy information about studies with human participants or human data. See also policy information about sex, gender (identity/presentation), and sexual orientation and race, ethnicity and racism.

Reporting on sex and gender	Not applicable
Reporting on race, ethnicity, or other socially relevant groupings	Not applicable
Population characteristics	Not applicable
Recruitment	Not applicable
Ethics oversight	Not applicable

Note that full information on the approval of the study protocol must also be provided in the manuscript.

Field-specific reporting

Please select the one below that is the best fit for your research. If you are not sure, read the appropriate sections before making your selection.

☒ Life sciences ☐ Behavioural & social sciences ☐ Ecological, evolutionary & environmental sciences

For a reference copy of the document with all sections, see nature.com/documents/nr-reporting-summary-flat.pdf

Life sciences study design

All studies must disclose on these points even when the disclosure is negative.

Sample size	The sample sizes for cryo-EM datasets were chosen as a practical size for data collection and processing and for sufficient resolution to discuss the structure. No statistical methods were used to predetermine sample sizes.
Data exclusions	cryo-EM data were excluded by 2D classification and heterogeneous refinement. For Fig. 4c, all of the datasets from reconstituted proteoliposomes that exhibited non-reproducible results giving outlying data points were excluded.
Replication	For cryo-EM data, three datasets were collected, processed individually and merged. For Fig 3a, each sample was derived from a single purification batch, and the experiment was independently repeated three times with similar results. A single series of diluted proteins was loaded into a gel and subjected to SDS-PAGE analysis for Fig 3b. The results of three to four repeated experiments (from reconstitution to measurement) were used to determine the equilibrium pmf at each Q condition for Fig 4c. Sample sizes (n) from left to right are: 3, 3, 3, 4, 3 (WT) and 4, 3, 3, 4, 3 (δΔN-α) for Fig 4c.
Randomization	Randomization was not relevant to the analysis in this study as samples were not grouped.
Blinding	Blinding is not needed for cryo-EM analysis because classification was performed by computer classification.

Reporting for specific materials, systems and methods

We require information from authors about some types of materials, experimental systems and methods used in many studies. Here, indicate whether each material, system or method listed is relevant to your study. If you are not sure if a list item applies to your research, read the appropriate section before selecting a response.

Materials & experimental systems

n/a	Involved in the study	
☒	☐	Antibodies
☒	☐	Eukaryotic cell lines
☒	☐	Palaeontology and archaeology
☒	☐	Animals and other organisms
☒	☐	Clinical data
☒	☐	Dual use research of concern
☒	☐	Plants

Methods

n/a	Involved in the study	
☒	☐	ChIP-seq
☒	☐	Flow cytometry
☒	☐	MRI-based neuroimaging

Plants

Seed stocks	Not applicable
Novel plant genotypes	Not applicable
Authentication	Not applicable
